# Self-reports vs. physical measures of spinal stiffness

**DOI:** 10.7717/peerj.9598

**Published:** 2020-12-07

**Authors:** Jonas Nielsen, Casper Glissmann Nim, Søren O’Neill, Eleanor Boyle, Jan Hartvigsen, Gregory N. Kawchuk

**Affiliations:** 1 Department of Sports Science and Clinical Biomechanics, University of Southern Denmark, Odense, Denmark; 2 Spinecenter of Southern Denmark, Lillebaelt Hospital, Middelfart, Denmark; 3 Institute of Regional Health Science, University of Southern Denmark, Odense, Denmark; 4 Nordic Institute of Chiropractic and Clinical Biomechanics, Odense, Denmark; 5 Department of Physical Therapy, Faculty of Rehabilitation Medicine, University of Alberta, Edmonton, Canada

**Keywords:** Vertetrack, Lumbar spine instability questionnaire, Lumbar stiffness disability index, Spinal stiffness, Low back pain, LSIQ, LSDI, LBP, P-A spinal stiffness measures, Self-reports

## Abstract

**Background:**

Objectively measured reduction in lumbar posterior-to-anterior (PA) stiffness is associated with pain relief in some, but not all persons with low back pain. Unfortunately, these measurements can be time consuming to perform. In comparison, the Lumbar Spine Instability Questionnaire (LSIQ) is intended to measure spinal instability and the Lumbar Spine Disability Index (LSDI) is created for self-reporting functional disability due to increased spinal stiffness. Given the above, the aim of this study is to compare measures of the LSIQ and LSDI with objective measures of lumbar PA stiffness as measured by a mechanical device, Vertetrack (VT), in patients with persistent non-specific low back pain (nsLBP).

**Methods:**

Twenty-nine patients with nsLBP completed the LSIQ and LSDI at baseline and after two weeks. On these same occasions, PA spinal stiffness was measured using the VT. Between measurements, patients received four sessions of spinal manipulation. The resulting data was analyzed to determine the correlation between the self-report and objective measures of stiffness at both time points. Further, the patients were categorized into responders and non-responders based on pre-established cut points depending on values from the VT and compared those to self-report measures in order to determine whether the LSIQ and the LSDI were sensitive to change.

**Results:**

Twenty-nine participants completed the study. Measures from the LSIQ and LSDI correlated poorly with objectively measured lumbar PA stiffness at baseline and also with the change scores. The change in objectively measured lumbar PA stiffness following spinal manipulation did not differ between those who improved, and those who did not improve according to the pre-specified cut-points. Finally, a reduction in lumbar PA stiffness following intervention was not associated with improvement in LSIQ and LSDI outcomes.

**Conclusions:**

The current data indicate that the LSIQ and LSDI questionnaires do not correlate with measures obtained objectively by VT. Our results suggest that these objective and self- reported measures represent different domains and as such, cannot stand in place of one another.

## Introduction

Low back pain (LBP) is the primary cause of years lived with disability globally (Haldeman et al., 2012; Collaborators GB of of DS 2013, 2015). No specific nociceptive source can be identified in the majority of these cases, and they are therefore classified as non-specific (ns) (Hartvigsen et al., 2018). Theoretically, classifying patients with nsLBP into subgroups based on clinical characteristics may generate better treatment outcomes (Wong & Kawchuk, 2016; Flynn et al., 2002). Clinical assessment of segmental spinal stiffness is one way of subdividing patients, which is often used by practitioners of spinal manipulation (SM) to decide where to apply treatment (Fritz, Whitman & Childs, 2005; Tuttle, 2009). However, manual assessment of segmental spinal stiffness has relatively poor intra- and interrater reliability, and therefore numerous devices have been developed for obtaining quantified measures of spinal stiffness, albeit mainly for research use, and the reported test-retest reliability of these devices is generally high (Wong & Kawchuk, 2016). One such device is the Vertetrack (VT) (Brown et al., 2017). The VT has produced reliable measurements quantifying the load–displacement values for within-session and between-session assessments in asymptomatic patients (Hadizadeh, Kawchuk & Parent, 2019; Wong et al., 2013), and it has demonstrated a high level of accuracy in a recent validation study (Young, 2019).

Patients with nsLBP display greater average lumbar posterior-to-anterior (PA) stiffness as measured by mechanical devices than asymptomatic people (Kawchuk et al., 2015). Objectively measured reduction in lumbar PA stiffness is associated with pain relief in some, but not all persons with nsLBP (Wong & Kawchuk, 2016). A decrease in stiffness has not previously been shown to occur in an asymptomatic cohort following spine mobilisation (Allison et al., 2001).

Spinal manipulation (SM) has been shown to alter lumbar PA stiffness measures, and a reduction in stiffness is related to self-reported measures of disability (Stanton et al., 2017; Wong et al., 2015). Feeling stiff in the lower back is reported to be a predictor of disability (Thakral et al., 2014) and a primary target in interventions for many musculoskeletal conditions including LBP (Stanton et al., 2017). Further studies are needed to obtain better insight into how measures of spinal stiffness relate to clinical practice. Specifically, there are now objective measures of lumbar spinal stiffness available but they are time consuming to perform and it is not yet clear how such measures relate to a number of clinically relevant self-reported outcomes (Wong & Kawchuk, 2016). Two such potentially relevant self-reported outcome instruments are the Lumbar Spine Instability Questionnaire (LSIQ) and the Lumbar Stiffness Disability Index (LSDI).

Recently, the clinimetric properties of the LSIQ were assessed in a sample of patients with nsLBP (Saragiotto et al., 2018). The authors found that the LSIQ has acceptable test-retest reliability, but also concluded that it remains unclear whether the LSIQ measures clinical instability or some other construct in nsLBP. Furthermore, the LSIQ was also reported to show poor internal consistency and unclear construct validity. Additional clarification of the underlying concepts of the LSIQ has therefore been advocated. Still, the measure has been used in clinical studies. Individuals that report feeling more unstable (compared with those that feel less unstable)—and who have higher scores of the LSIQ—have reported better outcomes from a motor control exercise intervention, compared to those completing a graded activity program (Macedo et al., 2014). Therefore, while the LSIQ is intended to measure instability, it has not been validated against direct measures of instability therefore opening the possibility that it may indeed reflect other biomechanical measures such as stiffness.

The LSDI has demonstrated acceptable internal consistency, retest reliability, and external validity when assessed in a group of 32 adult lumbar arthrodesis patients (Hart et al., 2013a). Also, increased patient-reported difficulty in performing activities of daily living (ADL), as indicated by a higher LSDI score, is correlated strongly with decreased lumbar range of motion as measured on flexion-extension lateral radiographs (Hart et al., 2013a).

Given the above, the aims of this study were to examine:

1. How measures from the LSIQ, LSDI and the VT change following the provision of SM in patients with persistent nsLBP.
2. How baseline measures from the LSIQ and LSDI correlate to baseline values of lumbar PA stiffness as measured by the VT.
3. How changes in LSIQ and LSDI measures correlate to potential changes in VT measures following SM intervention.
4. If there was a difference in VT change scores following SM intervention between those who responded and those not responding according to LSIQ and LSDI measures following SM intervention.
5. If a reduction in lumbar PA stiffness was associated with improving in LSIQ and LSDI measures following SM intervention.

## Materials & Methods

### Participants

Patients with persistent low back pain were recruited from the Spine Centre of Southern Denmark, a large regional hospital department with specialist focus on spinal pain syndromes, located in Middelfart, Denmark. Patients were referred to the department from primary practice (general medical practitioners, chiropractors, medical consultants) and other hospitals in the region. See Table 1 for inclusion and exclusion criteria. Research design: A clinical trial with repeated measures.

**Table 1:** Inclusion & exclusion criteria.

*To be enrolled in the study, the participant had to:*
∘ Fulfil informed written consent.
∘ Have the ability to speak and read Danish.
∘ Be between the age of 18 and 60.
∘ Have a body mass index <35
∘ Have had LBP >3 months, defined as pain on the posterior aspect of the body between the 12th thoracic vertebrae and the gluteal folds.
∘ Have no previous back surgery and not have had surgery in general in the last 4 months.
∘ Have received no spinal manipulation in the last month.
∘ Take no other pain medication than paracetamol, NSAIDs or weak synthetic opioids
∘ Have no competing diagnoses which could
(a) confound the diagnosis of nsLBP e.g., osteoporosis, cancer, fibromyalgia etc.
(b) interfere with the allocated treatment
*Participants were excluded during the study if they*:
∘ Were not completing the allocated intervention (minimum 75% of scheduled treatments).
∘ Did not fill out the questionnaires
∘ Received other treatment than that administered as part of the study.
∘ Deviated from the agreed upon medication at baseline measures within the treatment period.
∘ Were unable to hold breath for 10 s.

### Questionnaires

**Figure 1:**
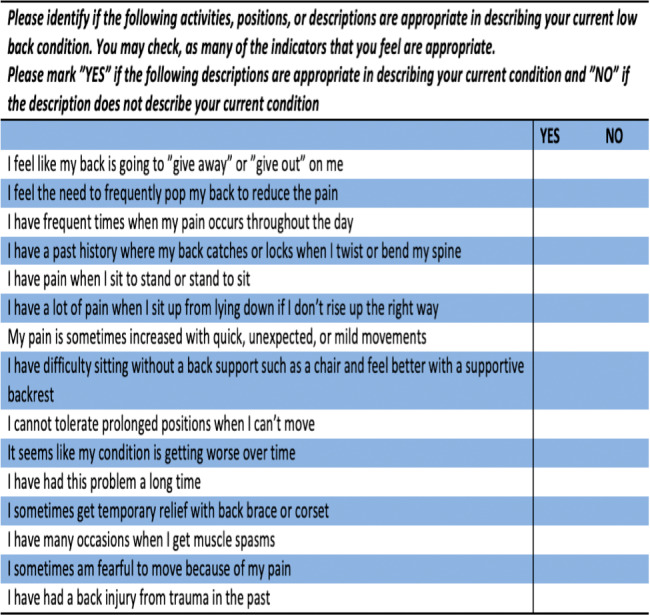
Lumbar Spine Instabilité Questionnaire (LSIQ).

The LSIQ is a self-report measure consisting of 15 items where higher scores are assumed to indicate greater clinical lumbar instability (Fig. 1). A single point is given for every “*yes*” answer, thus the score of the LSIQ ranges from 0–15 (Cook, Brismée & Sizer, 2006).

**Figure 2:**
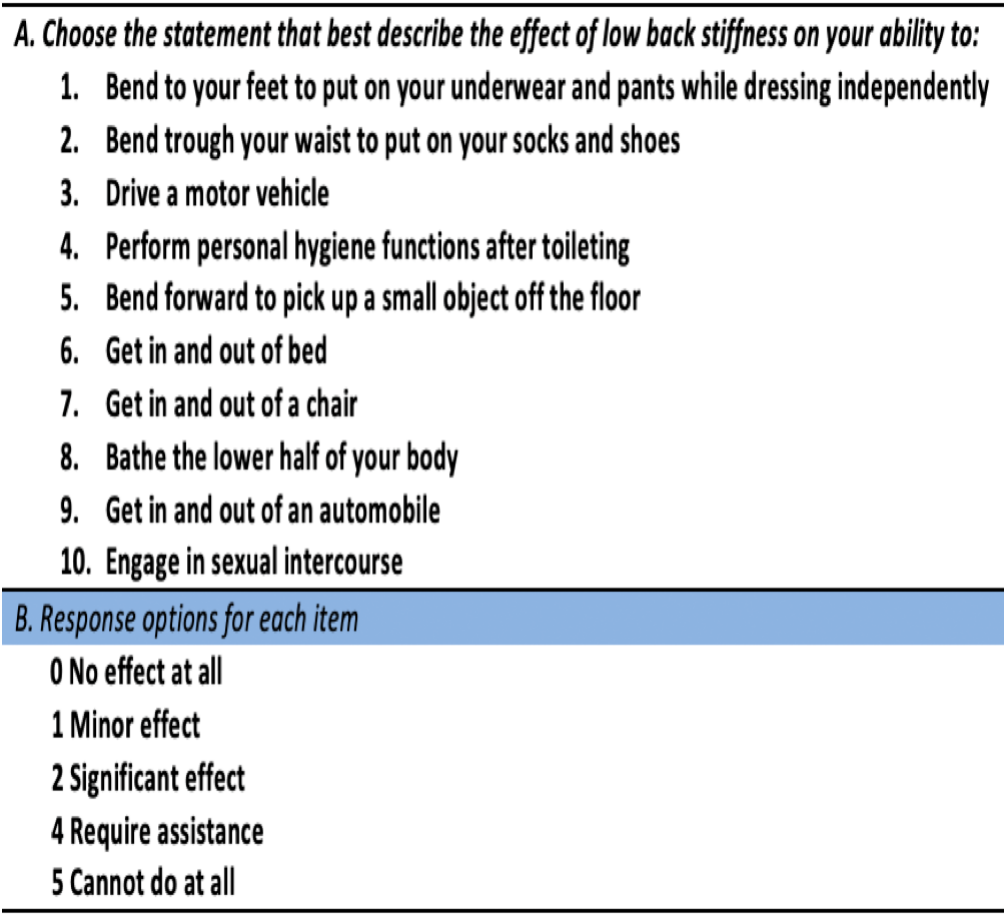
Lumbar Spine Disability Index (LSDI).

The LSDI consists of 10 items assessing the impact of low back stiffness on ADL such as dressing, hygiene, mobility, and sexual activity (Fig. 2). Responses to each item are scored from 0 (“No effect at all”) to 4 (“I cannot do this at all”). The raw score of the LSDI ranges from 0–40, and a percentage score is calculated from the raw score. Higher scores indicate greater disability due to stiffness (Hart et al., 2013a).

An English-Danish bi-lingual clinician at the Spine Centre experienced in the translation process of questionnaires performed the translation of both questionnaires into Danish. Demographic data was obtained from the SpineData questionnaire (Kent et al., 2015). This questionnaire is used at the hospital and has general questions about demographics, pain intensity, duration etc.

### Vertetrack measurements

**Figure 3:**
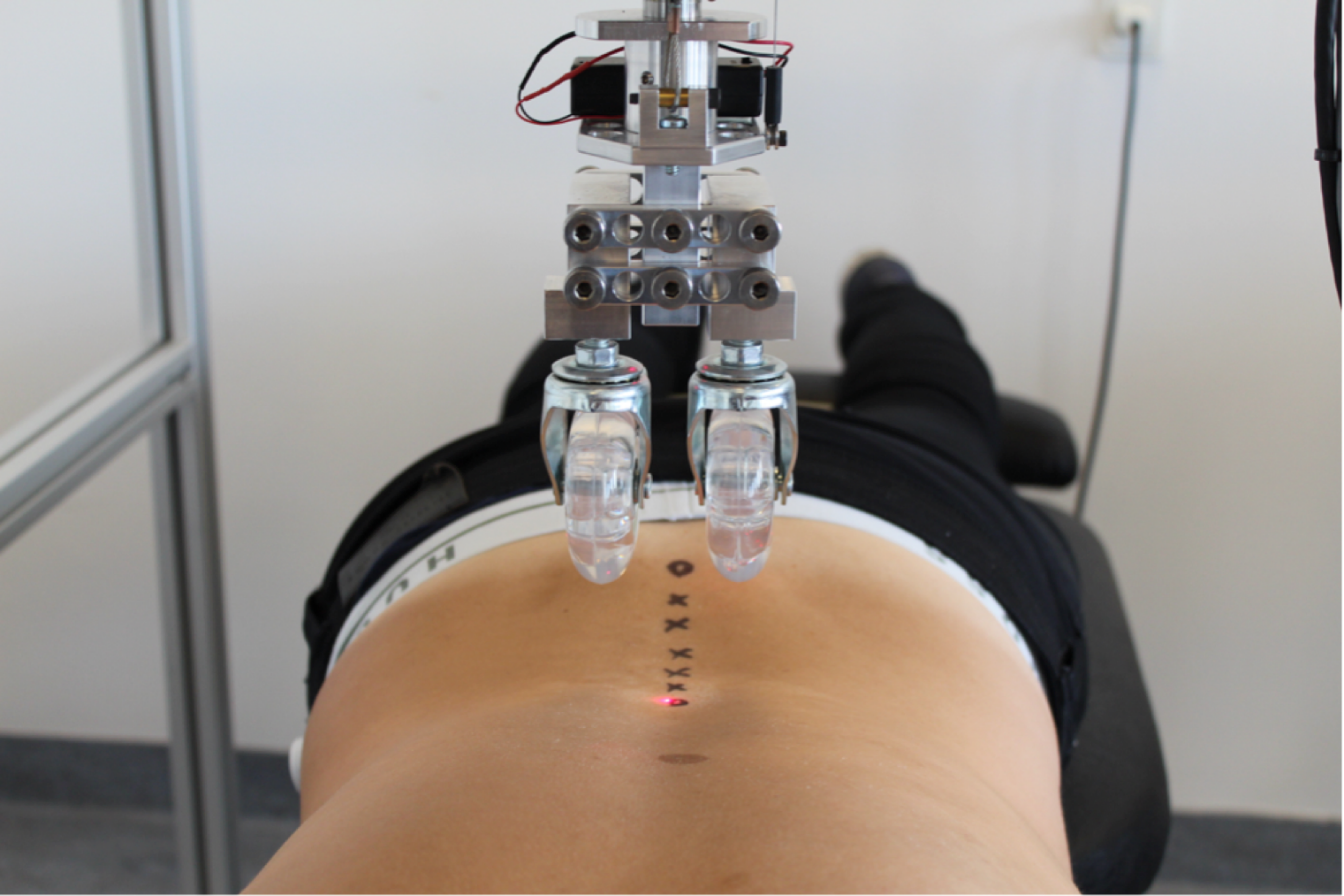
The Vertetrack with contact rollers and trajectory points.

The VT applies a pre-selected vertical load continuously over a specific spinal region (Brown et al., 2017). It consists of a solid, aluminum gantry on lockable caster wheels that can be positioned over a participant lying in the prone position on a standard plinth (Fig. 3). The frame is used to provide a rigid support for the indenter apparatus, which applies a vertical load to the region of interest. The indenter apparatus consists of a loading rod suspended within a linear bearing to permit near-frictionless vertical translation as the load is moved along the spine on a pair of roller wheels (diameter 70 mm, width 30 mm). These wheels straddle the midline either side of the test subject’s spinous processes, thus providing a rolling contact point for the application of PA loads. During application, various sensors measure the tissue deformation from the applied load as well as the position of the indenter along the spine. Using this setup, it is possible to position the indenter apparatus at defined waypoints along the spine, and then by a number of stepper motors have the indenter apparatus follow this pre-defined trajectory whilst applying a fixed PA-load to the subject through the two roller wheels. The result is a continuous and real time quantification of the bulk deformation of any spinal region for a given mass over a defined trajectory. Using a series of fixed loads in 10 Newton increments, the force–deformation profile of the spinal region of interest can be produced.

In this study, waypoints were identified at each lumbar spinous process using ultrasound, and marked on the surface of the skin with an ink pen. During data collection, the participants were asked to fully exhale and hold their breath. The roller was lowered onto the participant’s back and set in motion to follow the pre-defined waypoints. The testing procedure lasted approximately 10 s, and when testing was complete, the stepping motor system retracted the load from the participants back.

Prior to inclusion, potential participants underwent the usual clinical diagnostic procedures at the Spine Centre, including an extensive ‘SpineData’ research questionnaire. Potential participants were invited to participate only if a diagnosis of persistent nsLBP had been established. Participants included in the study completed the LSIQ and LSDI at baseline, and were scheduled for lumbar PA stiffness testing in the VT thereafter. The same procedure was repeated at a two-week follow up session.

### Spinal manipulative therapy (SMT)

**Figure 4:**
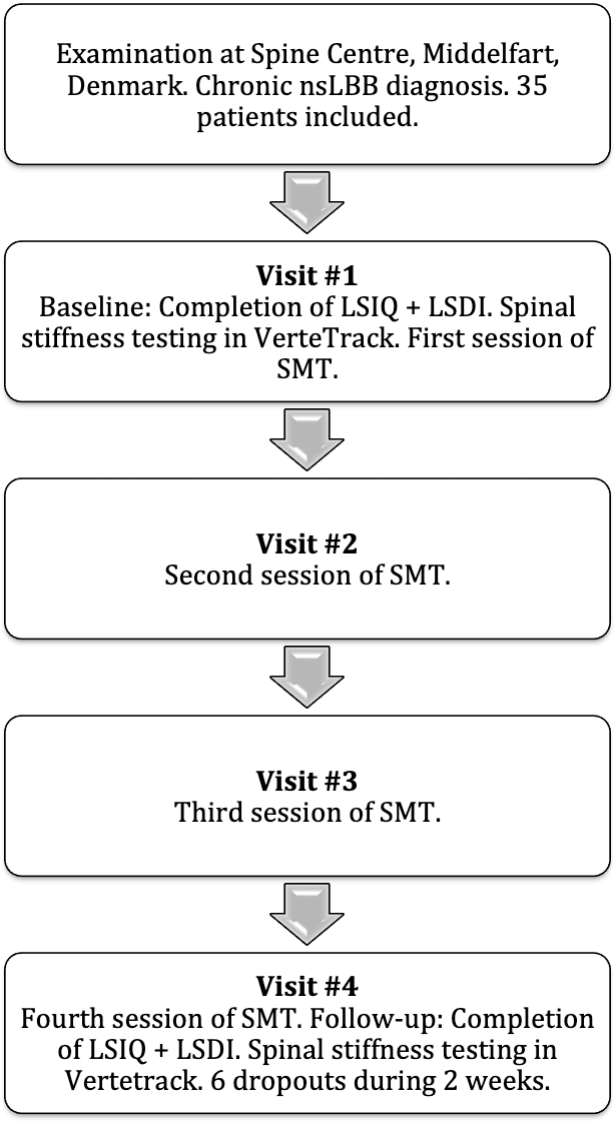
Study flow chart.

During the course of these two weeks, the participants were treated with spinal manipulation at the Spine Centre. The participants were placed in the side-position, and a standard manipulation lumbar-roll technique was applied (Thomas, Faculty & Clinic, (0000)). The intervention consisted of 4 treatment sessions of SM over a two-week period. The number of treatment sessions was chosen based on previous research, which reports that the majority of patients who improve with SM, will do so within four treatment sessions in the first two weeks (Stig, Nilsson & Leboeuf-Yde, 2001). The 4th session of SM was done within 5–10 min prior to the last VT follow-up session. See Fig. 4 for study flow diagram.

### Ethics

The project was conducted in accordance with the Helsinki-II declaration, and the project was approved by the Regional Committees on Health Research Ethics for Southern Denmark (S-20160201) and the Danish Data Protection Agency. ClinicalTrials.gov identifier: NCT04086667. All participants provided informed, written consent.

### Data analysis

Descriptive statistics were performed on clinical characteristics of the participants. Testing for normality for relevant variables was done using the Shapiro–Wilk’s test, all data was deemed normally distributed.

**Figure 5:**
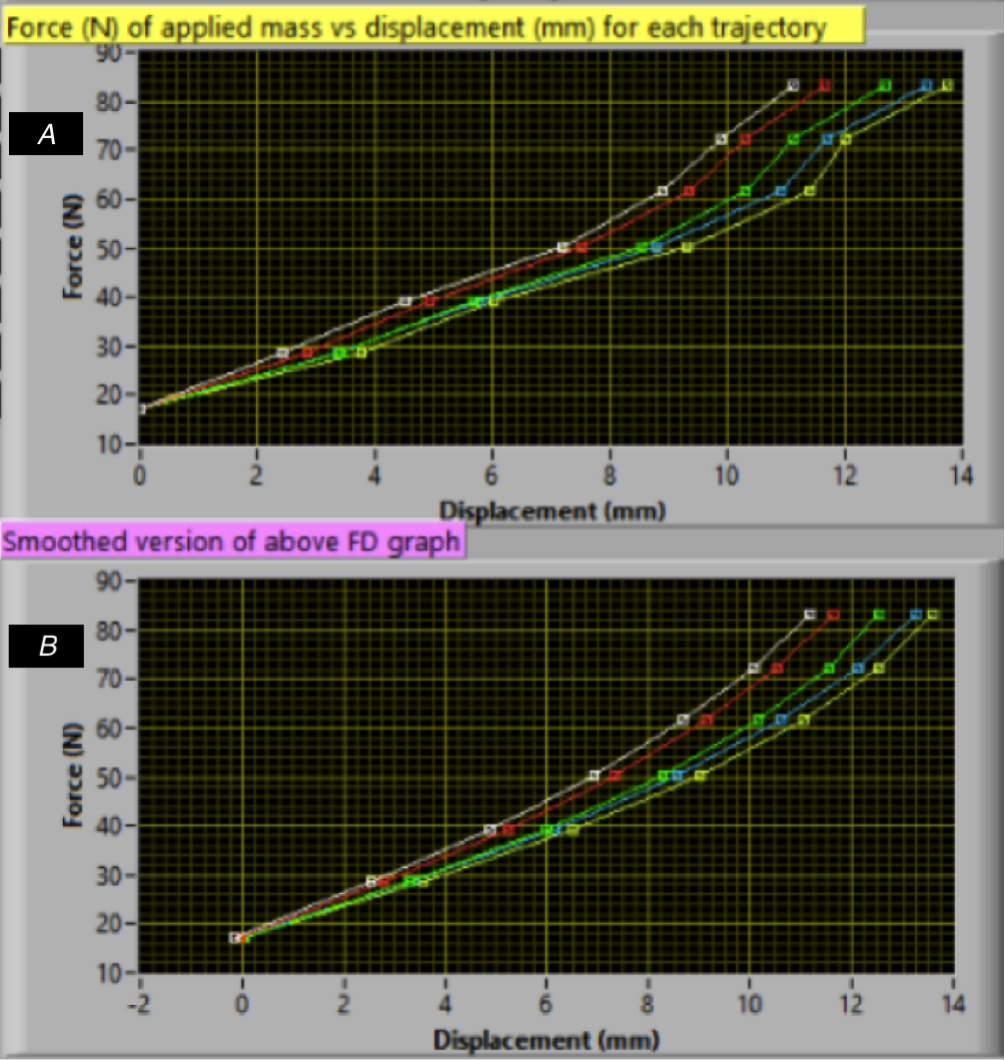
Force-displacement curve (A–B). Each point indicates added mass. (B) is a smoothed version of A. Raw data (A) was used to calculate the SS-scores.

Spinal stiffness data from the VT was categorized into *segmental stiffness* (SS), i.e., the individual stiffness scores of motion segments L1-L5, and *mean lumbar stiffness* (MLS), calculated as the mean of all SS-scores. Questionnaire data were treated as continuous. VT data were treated as continuous data. Specifically, SS measures were calculated from the second point to the second last point of the raw force–displacement curve (see Fig. 5). Hence, the SS measures equal the force (N) of the applied mass divided by the displacement (mm). The MLS was calculated as the mean of all five SS measures for each participant.

Paired *t*-tests were performed to examine the difference in questionnaire and VT measures before and after intervention.

Spearman’s analysis was performed to test the baseline correlation between measures of each questionnaire and the VT measures. Change scores from baseline to follow-up were calculated for SS, MLS and both questionnaires. Spearman’s analysis was again performed to test the correlation between change scores in measures from each questionnaire and change scores in the VT measures. Scatterplots were created to illustrate the relation between baseline and change score means of the questionnaires and VT measures.

Participants were classified as responders or non-responders to the SM intervention based on whether or not they achieved a decrease of two or more points in the LSIQ at follow-up compared to baseline. This was a predefined and arbitrary cut point, deemed relevant to our study only. Participants not achieving a two-point reduction were classified as non-responders.

This procedure was also performed for the LSDI, but here a cut point corresponding to a 12,5% (i.e., 5 out of 40 points) reduction in LSDI-scores at follow-up compared to baseline, classified participants as responders. This was based on a previous study reporting an 11% improvement (i.e., decrease) in LSDI-score following arthrodesis over a single lumbar segment.

A Welch two-sample *t*-test was performed to determine if there was a difference in the change scores of VT measures between the responders and non-responders, determined by the LSIQ and LSDI change respectively.

Finally, a 5% reduction in each VT measure (both SS and MLS) classified participants as responders. This specific cut point, which was also predefined and arbitrary, was considered relevant to our study only. A chi^2^ test was performed to determine whether a decrease in lumbar PA stiffness was associated with improvement in LSIQ and LSDI.

## Results

**Figure 6:**
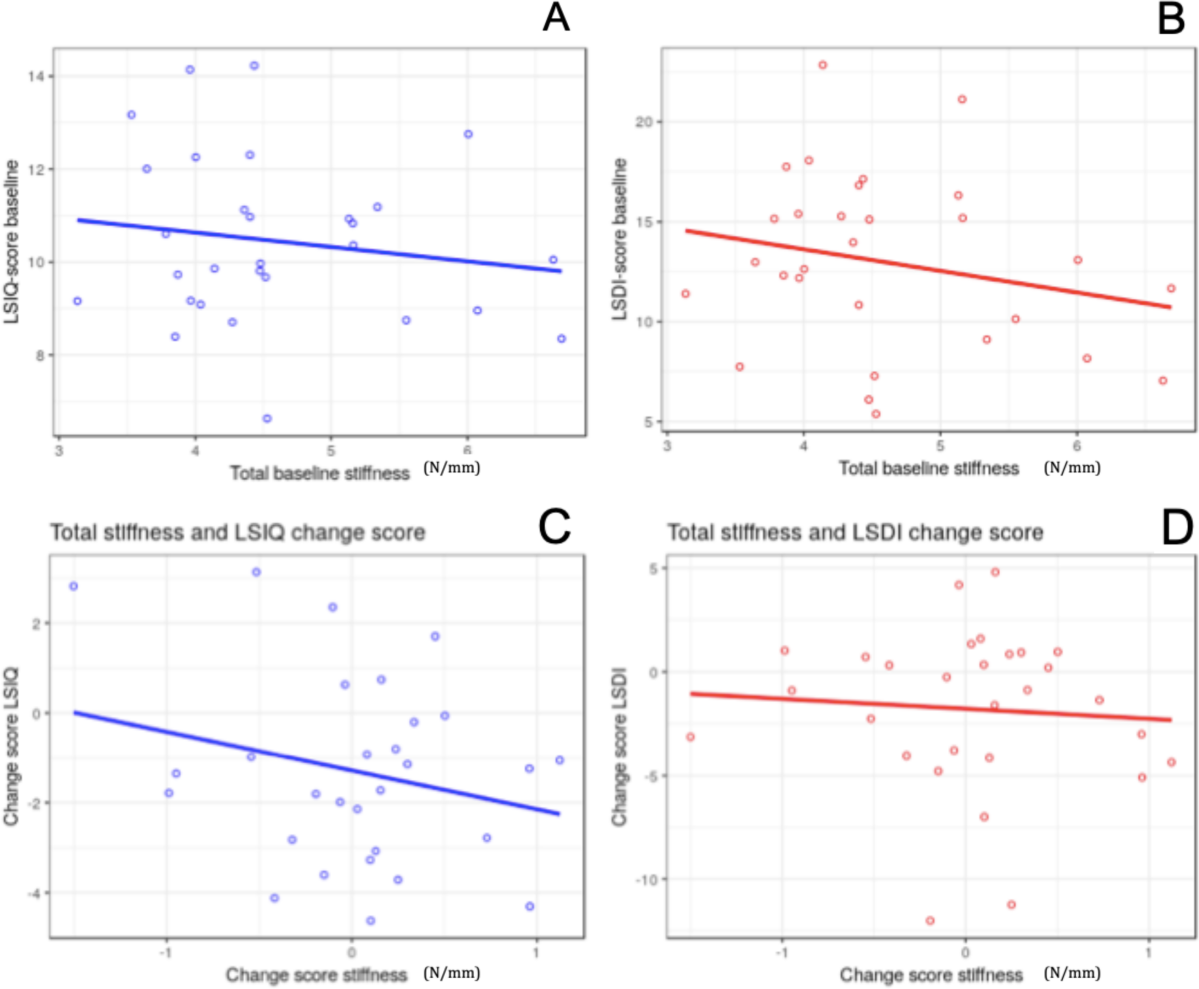
Scatterplots of linear relationships. (A) and (B) Relation between baseline LSIQ/LSDI scores and mean baseline stiffness score (N/mm). (C) and (D) Relation between LSIQ/LSDI change scores and stiffness change scores.

A total of 35 patients were recruited into the study (Table 2). Of those, 29 patients completed the full trial. All six dropouts missed two or more treatments during the two-week intervention period. Age, sex, duration of back pain and baseline back pain scores among the included 29 patients were not different from the 6 who did not complete the trial. From the resulting 29 participants, questionnaire and VT data were normally distributed at baseline and at follow-up. The change scores of the LSIQ and the SS change score for L1, L3, L4 and L5 were also normally distributed. The LSDI change score, the L2 change score and the MLS change score were not normally distributed. Figure 6 illustrates relations between measures from the LSIQ, LSDI and MLS.

**Table 2:** Clinical characteristics collected from the Spine Data questionnaire.

*Characteristics*	Mean (SD) *n* = 35
Age (years)	44.9 (9.9)
Male	22
Female	13
Duration of back pain (years)	5.2 (9.2)
Baseline back pain (NRS)	5.6 (2.0)

Table 3 presents the mean LSIQ, LSDI and VT measures at baseline, follow-up and for the change scores. From baseline to follow-up, the mean LSIQ and LSDI scores decreased by 1.3 (*P* = 0.003) and 4.5 (*P* = 0.018) points respectively. The MLS-score increased by 0.029 N/mm (*P* = 0.994).

**Table 3:** Results. LSIQ, LSDI and VT data (baseline, follow-up and change scores). Number of responders and non-responders including mean percentage change score following intervention.

	Baseline mean ± SD	Follow-up mean ± SD	Change score mean ± SD (*p*-value)	# Responders (mean % change score)	# Non-responders (mean % change score)
LSIQ	10.4 ± 1.7	9.1 ± 2.2	−1.3 ± 2.2 (0.003)	21 (−22.5)	8 (17.1)
LSDI	32.3 ± 11.0	27.8 ± 11.0	−4.5 ± 9.6 (0.018)	14 (−33.5)	15 (11.4)
VT(N/mm):					
L1	4.48 ± 0.96	4.48 ± 0.84	0.004 ± 0.73 (0.977)	10 (−15)	19 (10)
L2	4.50 ± 0.99	4.52 ± 0.84	0.025 ± 0.73 (0.854)	7 (−17.7)	22 (8.5)
L3	4.52 ± 0.93	4.57 ± 0.84	0.052 ± 0.59 (0.640)	9 (−12.5)	20 (8.7)
L4	4.63 ± 0.95	4.67 ± 0.84	0.042 ± 0.60 (0.711)	8 (−13.5)	21 (7.8)
L5	4.90 ± 0.99	4.92 ± 0.89	0.020 ± 0.68 (0.874)	10 (−12.8)	19 (9.2)
TLS	4.60 ± 0.91	4.63 ± 0.81	0.029 ± 0.58 (0.794)	7 (−14.7)	22 (6.7)

Twenty-one participants improved following the intervention period according to the pre-determined LSIQ cut-point, and 14 improved according to the LSDI-score (Table 3). Respectively, 10, 7, 9, 8 and 10 participants “improved” according to the segmental stiffness changes of L1-L5 (ie. a decrease in VT-scores). Seven participants improved as determined by the change in MLS. No statistically significant difference was found between responders and non-responders for either LSIQ or LSDI (Table 4).

**Table 4:** Results. Correlation between LSDI and SS/MLS, both at baseline and for the change scores. Welch two-sample *t*-test and chi-square test for association between responders/non-responders (see “data analysis”.

	Spearman: baseline rho (*p*-value)	Spearman: change score rho (*p*-value)	Welch: t-score (*p*-value)	Chi-squared (*p*-value)
LSIQ:				
L1	−0.08 (0.6634)	−0.16 (0.3964)	0.1081 (0.916)	0.04452 (0.8329)
L2	−0.12 (0.5226)	−0.14 (0.4695)	0.3500 (0.733)	0.00448 (0.9466)
L3	−0.15 (0.4443)	−0.07 (0.7026)	0.6307 (0.542)	0.21577 (0.6423)
L4	−0.12 (0.5319)	0.00 (0.9928)	0.8098 (0.434)	0.54350 (0.4610)
L5	−0.14 (0.4545)	−0.09 (0.6267)	0.9746 (0.347)	3.83900 (0.0501)
MLS	−0.17 (0.3736)	−0.06 (0.7578)	0.6106 (0.554)	0.00087 (0.9764)
LSDI:				
L1	−0.07 (0.7078)	−0.08 (0.6812)	0.5003 (0.621)	0.84020 (0.3593)
L2	−0.15 (0.4485)	−0.21 (0.2860)	0.9210 (0.366)	0.29054 (0.5899)
L3	−0.28 (0.1435)	−0.07 (0.7058)	0.7927 (0.436)	0.07672 (0.7818)
L4	−0.2 (0.2926)	0.00 (0.9827)	0.7670 (0.451)	0.01315 (0.9087)
L5	−0.24 (0.2139)	0.01 (0.9725)	0.5114 (0.614)	0.01817 (0.8928)
MLS	−0.23 (0.2352)	−0.06 (0.7677)	0.7887 (0.438)	0.27898 (0.5974)

A reduction in objectively measured lumbar PA stiffness following treatment was not associated with improvement in either the LSIQ or LSDI following the SM intervention (Table 4).

## Discussion

Improvement in both LSIQ and LSDI scores were observed in patients with persistent nsLBP following a two-week intervention of 4 sessions of SM. The mean lumbar PA stiffness measured by the VT increased slightly during the intervention period among all participants, however not significantly. Neither the cross-sectional measures nor the change scores of the LSIQ and LSDI, correlated with objectively measured lumbar PA stiffness as measured by the VT. Those improving in the LSIQ or LSDI scores following intervention did not differ in the change scores of objectively measured PA stiffness compared to those not improving in the questionnaires scores. Finally, a decrease in lumbar PA stiffness was not associated with improving in the LSIQ and LSDI outcome following SM intervention. These findings suggest that these subjective and objective measures do not measure similar domains.

Stanton et al. recently concluded that perceived and actual, measured stiffness did not correlate well and that the experience of feeling stiff did not reflect actual biomechanical back stiffness as measured also by the VT (Stanton et al., 2017). They also found no difference in objective spinal stiffness between those with and without reported stiffness and LBP. From this work, they concluded that bodily feelings of stiffness may reflect a multisensory perceptual inference that aids bodily protection and the conscious perception of stiffness may not be the result only from joint relevant sensory information. Therefore, our results support the interpretation that it may not be feasible to rely on self-report when assessing stiffness of the spine –and also probably not when assessing constructs such as instability. Our results support this interpretation. It seems difficult to achieve measures of low back perceptions from a questionnaire, and even harder to gain knowledge as to what degree these perceptions reflect pain-related biomechanical changes of low back stiffness.

There are several possibilities as to why the objective measure of spinal stiffness did not change during the duration of this study. Most likely, the subset of participants we tested were not responders to SMT. As mentioned in the introduction, prior work has shown that some, but not all, persons who respond to SMT with a significant change in disability also show a significant change in spinal stiffness. Clearly, as is the case with all studies, not every recruited participant will be a responder.

Findings from a recent study, suggests that the 15 items of the LSIQ may be indicative of clinical instability individually, but that many of the questionnaire items are characteristics of LBP in general (Saragiotto et al., 2018). Analysis of the differential item functioning (item bias) identified several items that were significantly and meaningfully biased by factors other than lumbar instability, which is the proposed construct of the questionnaire (Saragiotto et al., 2018). This creates the possibility that the LSIQ evaluates properties other than instability such as stiffness. Unfortunately, the questionnaire did not correlates with objectively measured lumbar PA stiffness in our study.

Interestingly, the mean LSDI scores in a study that examined patients with lumbar arthrodesis were similar to the mean baseline and follow-up scores found in our study (Hart et al., 2013b). In fact, the mean baseline LSDI score (=32,3) among the patients in our study, who did not undergo any spinal fusion, was almost as high as the score among 21 patients with fusion of five or more lumbar segments (=35,4), and even higher than the mean score among the 24 patients with one-level fusion (=24,2). Another study sample of lumbar fusion patients reported LSDI scores similar to the LSDI scores in our study (Hart et al., 2014). This raises the question whether stiffness, which according to the LSDI is implied to cause limitation of ADL, is really due to the segmental fusion. Furthermore, it raises the question whether the LSDI reflects perceptions of lumbar stiffness or some other construct. The LSDI may detect some kind of disability in performing ADLs, but it can be questioned whether this is truly because of the segmental stiffness created by spinal fusion. So, as with the case of the LSIQ, this could be part of the explanation of a poor relationship between the self-reported LSDI and the objective measure of stiffness from the VT. In addition, our findings may also reflect that the VT quantifies neutral zone spine stiffness while the questions on the LSDI are directed at ranges of motion beyond the neutral zone.

### Study strengths

This study examined the relation between self-reported and objectively measured spinal stiffness both cross-sectionally and longitudinally, which expanded our assessment to include the association of the potential changes that might have occurred both in the questionnaire scores and measured stiffness. In addition, the self-report instruments were bench marked against an objective measure of spinal stiffness.

### Study limitations

The fact that translation of the questionnaires into Danish was not performed using the recommended reverse translation procedures might have an influence on the reliability of the conclusions from this study. Another limitation to this study is that division into improvement/no improvement in the measures of the LSIQ, LSDI and VT following intervention were set by our estimate of a relevant cut point. However, this was a necessary procedure because of the lack of previously utilizable cut points in the literature. Further, a number of factors can potentially influence the PA spinal stiffness measured by the VT. Voluntary/involuntary paraspinal muscle activation is one example. As for discussion of the limitations and challenges of the VT we refer to previous work (Wong & Kawchuk, 2016; Wong et al., 2013). Finally, it is possible that any effects of spinal manipulation could be attenuated by not taking the participants’ stiffness and/or instability status at baseline into consideration.

## Conclusions

The current data indicate, that the LSIQ and LSDI questionnaires do not measure actual spinal stiffness, in so much as this is quantified objectively by VT. Our results suggest that objective and self- reported measures of stiffness represent different domains. The questionnaires may still be of clinical relevance, but it seems they are unsuited as measures of PA spinal stiffness.

##  Supplemental Information

10.7717/peerj.9598/supp-1 Supplemental Information 1 Codes from ”R”

10.7717/peerj.9598/supp-2 Supplemental Information 2 Consort Checklist

10.7717/peerj.9598/supp-3 Supplemental Information 3 Raw data

10.7717/peerj.9598/supp-4 Supplemental Information 4 LSIQ & LSDI (Danish version)

## Additional Information and Declarations

### Data Availability

The following information was supplied regarding data availability: Raw data and code are available as Supplementary Files.

### Clinical Trial Registration

The following information was supplied regarding Clinical Trial registration: NCT04086667

## References

[ref-1] Allison G, Edmonston S, Kiviniemi K, Lanigan H, Simonsen AV, Walcher S (2001). Influence of standardized mobilization on the posteroanterior stiffness of the lumbar spine in asymptomatic subjects. Physiotherapy Research International.

[ref-2] Brown BT, Blacke A, Carroll V, Graham PL, Kawchuk G, Downie A (2017). The comfort and safety of a novel rolling mechanical indentation device for the measurement of lumbar trunk stiffness in young adults. Chiropractic & Manual Therapies.

[ref-3] Cook C, Brismée JM, Sizer PS (2006). Subjective and objective descriptors of clinical lumbar spine instability: a Delphi study. Manual Therapy.

[ref-4] Collaborators GB of DS (2015). Global, regional, and national incidence, prevalence, and years lived with disability for 301 acute and chronic diseases and injuries in 188 countries, 19902013: a systematic analysis for the Global Burden of Disease Study 2013. Lancet.

[ref-5] Flynn T, Fritz J, Whitman J, Wainner R, Magel J, Rendeiro D (2002). A clinical prediction rule for classifying patients with low back pain who demonstrate short-term improvement with spinal manipulation. Spine.

[ref-6] Fritz JM, Whitman JM, Childs JD (2005). Lumbar spine segmental mobility assessment: an examination of validity for determining intervention strategies in patients with low back pain. Archives of Physical Medicine and Rehabilitation.

[ref-7] Hadizadeh M, Kawchuk GN, Parent E (2019). Reliability of a new loaded rolling wheel system for measuring spinal stiffness in asymptomatic participants. BMC Musculoskeletal Disorders.

[ref-8] Haldeman S, Kopansky-Giles D, Hurwitz EL, Hoy D, Erwin WMark, Dagenais S (2012). Advancements in the management of spine disorders. Best Practice & Research. Clinical Rheumatology.

[ref-9] Hart RA, Gundle KR, Pro SL, Marshall LM (2013a). Lumbar Stiffness Disability Index: pilot testing of consistency, reliability, and validity. Spine Journal.

[ref-10] Hart RA, Marshall LM, Hiratzka SL, Kane MS, Volpi J, Hiratzka JR (2014). Functional limitations due to stiffness as a collateral impact of instrumented arthrodesis of the lumbar spine. Spine.

[ref-11] Hart RA, Pro SL, Gundle KR, Marshall LM (2013b). Lumbar stiffness as a collateral outcome of spinal arthrodesis: a preliminary clinical study. Spine Journal.

[ref-12] Hartvigsen J, Hancock MJ, Kongsted A, Louw Q, Ferreira ML, Genevay S, Hoy D, Karppinen J, Pransky G, Sieper J, Smeets RJ, Underwood M, Lancet Low Back Pain Series Working Group (2018). What low back pain is and why we need to pay attention. The Lancet.

[ref-13] Kawchuk GN, Edgecombe TL, Wong AYL, Cojocaru A, Prasad N (2015). A non-randomized clinical trial to assess the impact of nonrigid, inelastic corsets on spine function in low back pain participants and asymptomatic controls. Spine Journal.

[ref-14] Kent P, Kongsted A, Jensen TS, Albert HB, Schiøttz-Christensen B, Manniche C (2015). Spine Data –a Danish clinical registry of people with chronic back pain. Journal of Clinical Epidemiology.

[ref-15] Macedo LG, Maher CG, Hancock MJ, Kamper SJ, McAuley JH, Stanton TR (2014). Predicting response to motor control exercises and graded activity for patients with low back pain: preplanned secondary analysis of a randomized controlled trial. Physical Therapy.

[ref-16] Saragiotto BT, Maher CG, New CH, Catley M, Hancock MJ, Cook CE, Hodges PW (2018). Clinimetric testing of the lumbar spine instability questionnaire. Journal of Orthopaedic & Sports Physical Therapy.

[ref-17] Stanton TR, Moseley GL, Wong AYL, Kawchuk GN (2017). Feeling stiffness in the back: a protective perceptual inference in chronic back pain. Scientific Reports.

[ref-18] Stig LC, Nilsson O, Leboeuf-Yde C (2001). Recovery pattern of patients treated with chiropractic spinal manipulative therapy for long-lasting or recurrent low back pain. Journal of Manipulative and Physiological Therapeutics.

[ref-19] Thakral M, Shi L, Shmerling RH, Bean JF, Leveille SG (2014). A stiff price to pay: does joint stiffness predict disability in an older population?. Journal of the American Geriatrics Society.

[ref-20] Thomas F, Faculty C, Clinic C Chiropractic technique principles and procedures.

[ref-21] Tuttle N (2009). Is it reasonable to use an individual patient’s progress after treatment as a guide to ongoing clinical reasoning?. Journal of Manipulative and Physiological Therapeutics.

[ref-22] Wong AYL, Kawchuk GN (2016). The clinical value of assessing lumbar posteroanterior segmental stiffness: a narrative review of manual and instrumented methods. PM & R.

[ref-23] Wong AYL, Kawchuk G, Parent E, Prasad N (2013). Within- and between-day reliability of spinal stiffness measurements obtained using a computer controlled mechanical indenter in individuals with and without low back pain. Manual Therapy.

[ref-24] Wong AYL, Parent EC, Dhillon SS, Prasad N, Kawchuk GN (2015). Do participants with low back pain who respond to spinal manipulative therapy differ biomechanically from nonresponders. Untreated controls or asymptomatic controls?. Spine.

[ref-25] Young A (2019). Validating assessment of spinal stiffness: bench-top performance of the VerteTrack system. Masters Res thesis.

