# Supplementary material for: Self-reports vs. physical measures of spinal stiffness
Source: PeerJ. 2020 Dec 7;8:e9598. doi: 10.7717/peerj.9598 (PMC7727369; doi:10.7717/peerj.9598)
Supplement: Supplemental Information 1 [file peerj-08-9598-s001.docx]

Analysis, code materials

# Test for normality

**#Test of normality (stiffness pre)**

ggplot(stif_c,aes(L1.pre)) +
 geom_histogram()

## `stat_bin()` using `bins = 30`. Pick better value with `binwidth`.


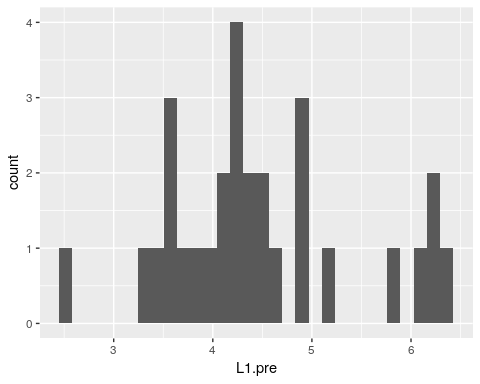


shapiro.test(stif_c$L1.pre)

##
## Shapiro-Wilk normality test
##
## data: stif_c$L1.pre
## W = 0.9362, p-value = 0.07976

ggplot(stif_c,aes(L2.pre)) +
 geom_histogram()

## `stat_bin()` using `bins = 30`. Pick better value with `binwidth`.


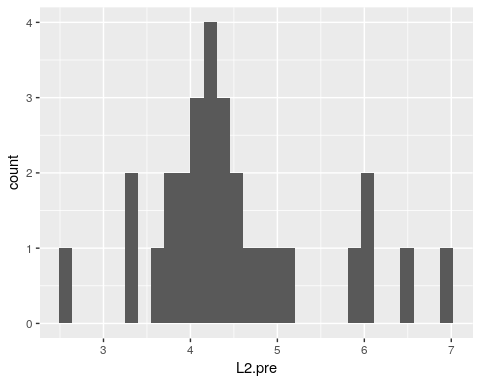


shapiro.test(stif_c$L2.pre)

##
## Shapiro-Wilk normality test
##
## data: stif_c$L2.pre
## W = 0.93253, p-value = 0.06399

ggplot(stif_c,aes(L3.pre)) +
 geom_histogram()

## `stat_bin()` using `bins = 30`. Pick better value with `binwidth`.


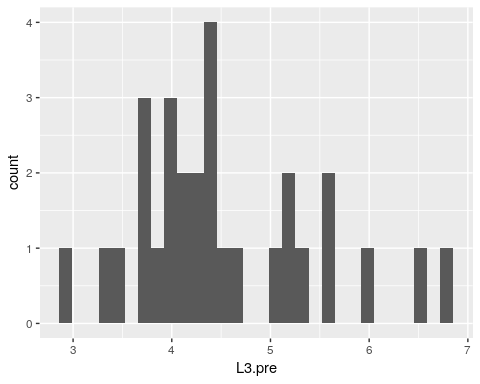


shapiro.test(stif_c$L3.pre)

##
## Shapiro-Wilk normality test
##
## data: stif_c$L3.pre
## W = 0.94634, p-value = 0.147

ggplot(stif_c,aes(L4.pre)) +
 geom_histogram()

## `stat_bin()` using `bins = 30`. Pick better value with `binwidth`.


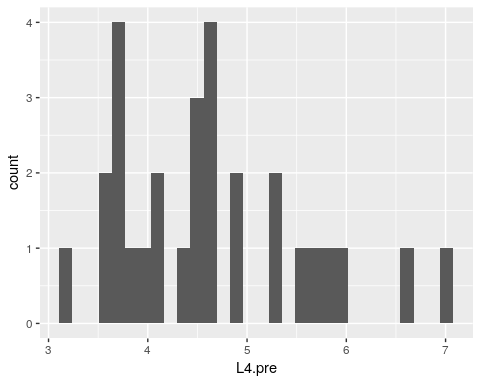


shapiro.test(stif_c$L4.pre)

##
## Shapiro-Wilk normality test
##
## data: stif_c$L4.pre
## W = 0.94049, p-value = 0.1033

ggplot(stif_c,aes(L5.pre)) +
 geom_histogram()

## `stat_bin()` using `bins = 30`. Pick better value with `binwidth`.


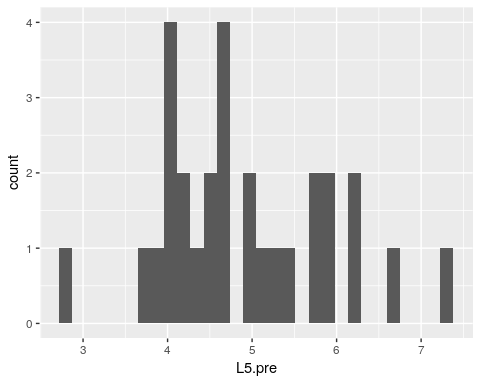


shapiro.test(stif_c$L5.pre)

##
## Shapiro-Wilk normality test
##
## data: stif_c$L5.pre
## W = 0.96953, p-value = 0.547

**#Test of normality (stiffness post)**

ggplot(stif_c,aes(L1.post)) +
 geom_histogram()

## `stat_bin()` using `bins = 30`. Pick better value with `binwidth`.


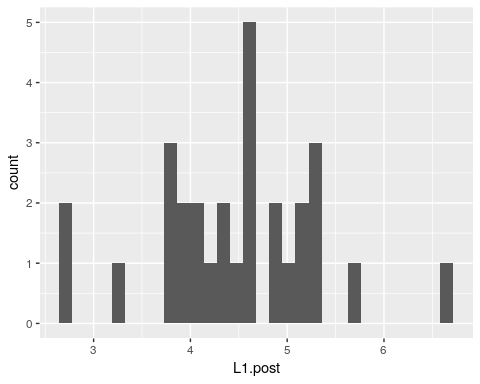


shapiro.test(stif_c$L1.post)

##
## Shapiro-Wilk normality test
##
## data: stif_c$L1.post
## W = 0.97526, p-value = 0.708

ggplot(stif_c,aes(L2.post)) +
 geom_histogram()

## `stat_bin()` using `bins = 30`. Pick better value with `binwidth`.


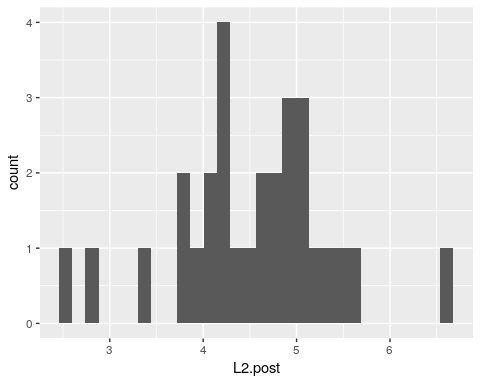


shapiro.test(stif_c$L2.post)

##
## Shapiro-Wilk normality test
##
## data: stif_c$L2.post
## W = 0.96982, p-value = 0.5548

ggplot(stif_c,aes(L3.post)) +
 geom_histogram()

## `stat_bin()` using `bins = 30`. Pick better value with `binwidth`.


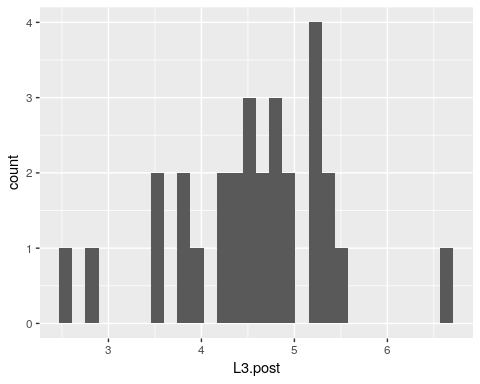


shapiro.test(stif_c$L3.post)

##
## Shapiro-Wilk normality test
##
## data: stif_c$L3.post
## W = 0.96772, p-value = 0.4997

ggplot(stif_c,aes(L4.post)) +
 geom_histogram()

## `stat_bin()` using `bins = 30`. Pick better value with `binwidth`.


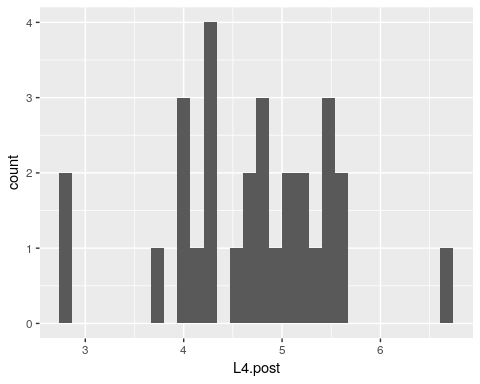


shapiro.test(stif_c$L4.post)

##
## Shapiro-Wilk normality test
##
## data: stif_c$L4.post
## W = 0.96665, p-value = 0.4729

ggplot(stif_c,aes(L5.post)) +
 geom_histogram()

## `stat_bin()` using `bins = 30`. Pick better value with `binwidth`.


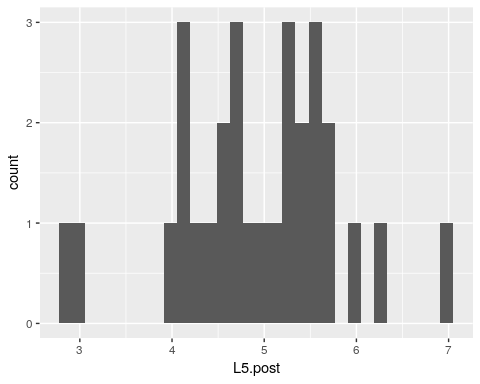


shapiro.test(stif_c$L5.post)

##
## Shapiro-Wilk normality test
##
## data: stif_c$L5.post
## W = 0.97413, p-value = 0.6757

**#Test of normality (stiffness change score)**

ggplot(stif_c,aes(L1.change)) +
 geom_histogram()

## `stat_bin()` using `bins = 30`. Pick better value with `binwidth`.


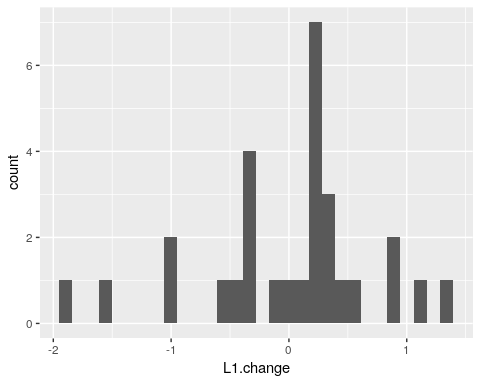


shapiro.test(stif_c$L1.change)

##
## Shapiro-Wilk normality test
##
## data: stif_c$L1.change
## W = 0.94038, p-value = 0.1026

ggplot(stif_c,aes(L2.change)) +
 geom_histogram()

## `stat_bin()` using `bins = 30`. Pick better value with `binwidth`.


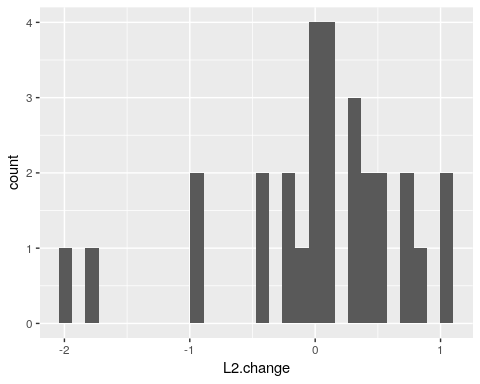


shapiro.test(stif_c$L2.change)

##
## Shapiro-Wilk normality test
##
## data: stif_c$L2.change
## W = 0.89606, p-value = 0.007893

ggplot(stif_c,aes(L3.change)) +
 geom_histogram()

## `stat_bin()` using `bins = 30`. Pick better value with `binwidth`.


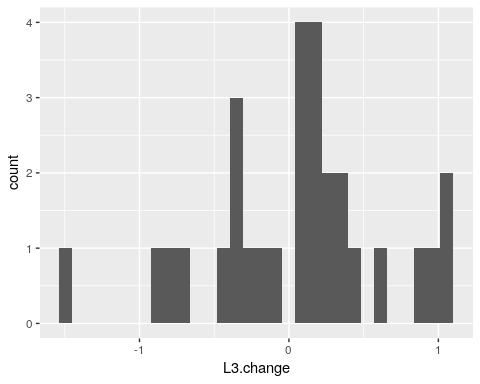


shapiro.test(stif_c$L3.change)

##
## Shapiro-Wilk normality test
##
## data: stif_c$L3.change
## W = 0.95819, p-value = 0.2964

ggplot(stif_c,aes(L4.change)) +
 geom_histogram()

## `stat_bin()` using `bins = 30`. Pick better value with `binwidth`.


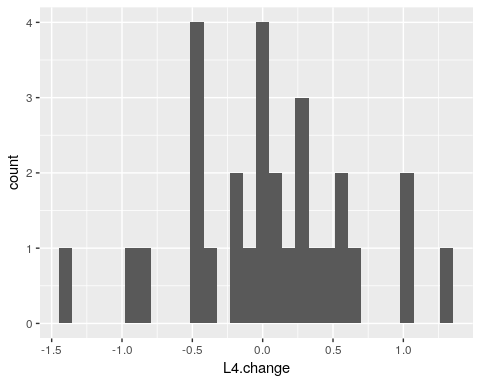


shapiro.test(stif_c$L4.change)

##
## Shapiro-Wilk normality test
##
## data: stif_c$L4.change
## W = 0.98412, p-value = 0.9283

ggplot(stif_c,aes(L5.change)) +
 geom_histogram()

## `stat_bin()` using `bins = 30`. Pick better value with `binwidth`.


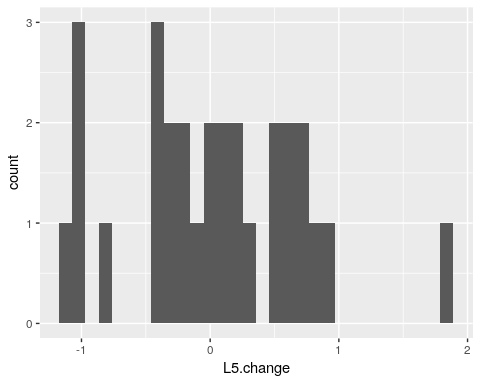


shapiro.test(stif_c$L5.change)

##
## Shapiro-Wilk normality test
##
## data: stif_c$L5.change
## W = 0.96569, p-value = 0.4495

**#Test of normality (LSIQ pre)**

ggplot(stif_c,aes(pre_LSIQ.total.score)) +
 geom_histogram()

## `stat_bin()` using `bins = 30`. Pick better value with `binwidth`.


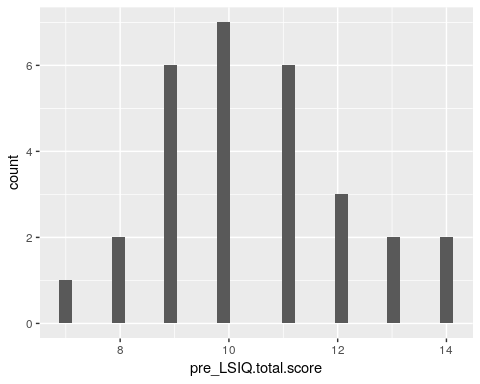


shapiro.test(stif_c$pre_LSIQ.total.score)

##
## Shapiro-Wilk normality test
##
## data: stif_c$pre_LSIQ.total.score
## W = 0.95769, p-value = 0.288

**#Test of normality (LSIQ post)**

ggplot(stif_c,aes(post_LSIQ.total.score)) +
 geom_histogram()

## `stat_bin()` using `bins = 30`. Pick better value with `binwidth`.


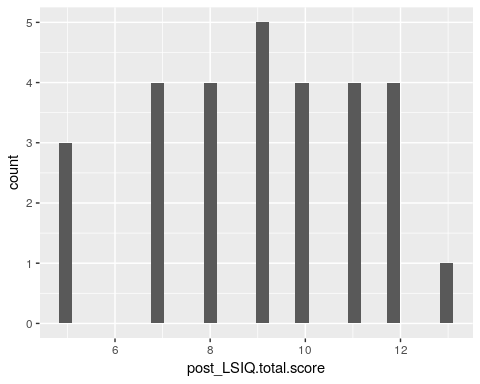


shapiro.test(stif_c$post_LSIQ.total.score)

##
## Shapiro-Wilk normality test
##
## data: stif_c$post_LSIQ.total.score
## W = 0.95219, p-value = 0.2085

**#Test of normality (LSIQ change score)**

ggplot(stif_c,aes(LSIQ.change.score)) +
 geom_histogram()

## `stat_bin()` using `bins = 30`. Pick better value with `binwidth`.


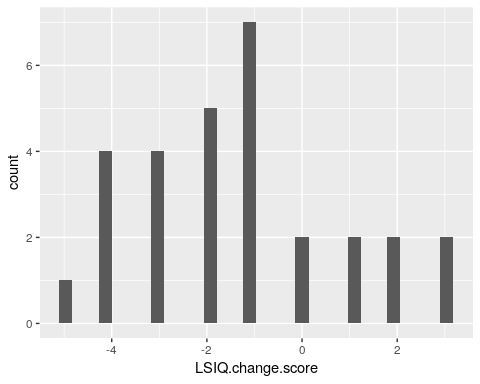


shapiro.test(stif_c$LSIQ.change.score)

##
## Shapiro-Wilk normality test
##
## data: stif_c$LSIQ.change.score
## W = 0.94868, p-value = 0.1691

**#Test of normality (LSDI pre)**

ggplot(stif_c,aes(pre_LSDI.total.score)) +
 geom_histogram()

## `stat_bin()` using `bins = 30`. Pick better value with `binwidth`.


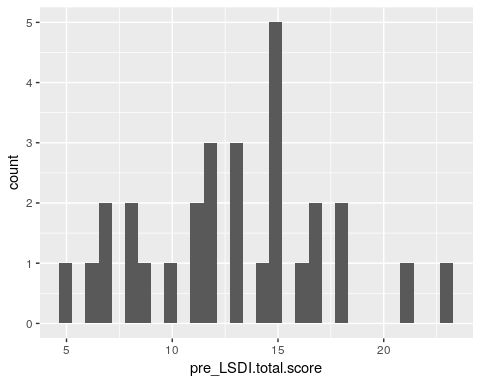


shapiro.test(stif_c$pre_LSDI.total.score)

##
## Shapiro-Wilk normality test
##
## data: stif_c$pre_LSDI.total.score
## W = 0.9797, p-value = 0.8306

**#Test of normality (LSDI post)**

ggplot(stif_c,aes(post_LSDI.total.score)) +
 geom_histogram()

## `stat_bin()` using `bins = 30`. Pick better value with `binwidth`.


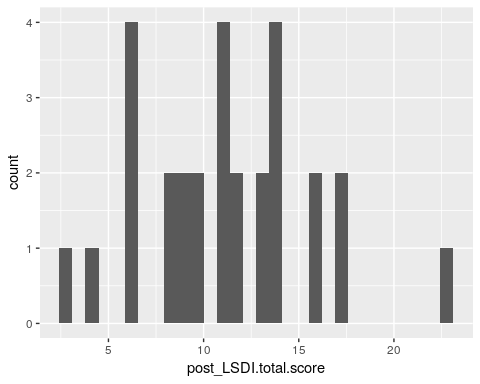


shapiro.test(stif_c$post_LSDI.total.score)

##
## Shapiro-Wilk normality test
##
## data: stif_c$post_LSDI.total.score
## W = 0.97438, p-value = 0.6829

**#Test of normality (LSDI change score)**

ggplot(stif_c,aes(LSDI.change.score)) +
 geom_histogram()

## `stat_bin()` using `bins = 30`. Pick better value with `binwidth`.


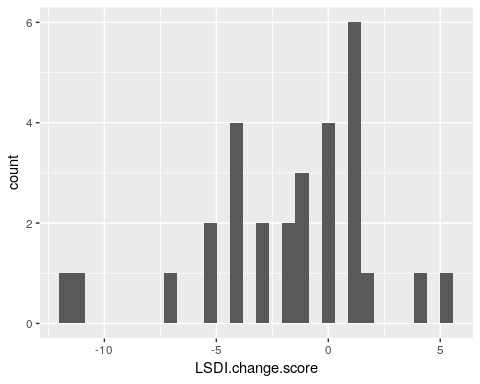


shapiro.test(stif_c$LSDI.change.score)

##
## Shapiro-Wilk normality test
##
## data: stif_c$LSDI.change.score
## W = 0.92771, p-value = 0.048

# LSIQ – change

#L1
stif.lm.L1.LSIQ<- lm(stif_c$L1.change~stif_c$LSIQ.change.score)
summary(stif.lm.L1.LSIQ)

##
## Call:
## lm(formula = stif_c$L1.change ~ stif_c$LSIQ.change.score)
##
## Residuals:
## Min 1Q Median 3Q Max
## -1.97436 -0.35636 0.08788 0.40001 1.41739
##
## Coefficients:
## Estimate Std. Error t value Pr(>|t|)
## (Intercept) -0.10139 0.15774 -0.643 0.526
## stif_c$LSIQ.change.score -0.08038 0.06332 -1.269 0.215
##
## Residual standard error: 0.7225 on 27 degrees of freedom
## Multiple R-squared: 0.05632, Adjusted R-squared: 0.02137
## F-statistic: 1.612 on 1 and 27 DF, p-value: 0.2151

plot(stif.lm.L1.LSIQ)


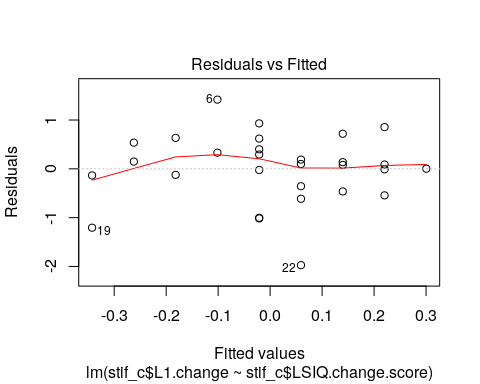

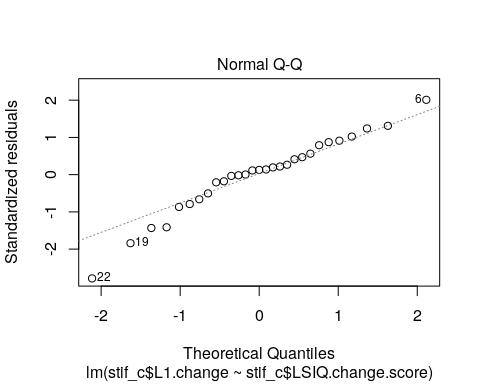

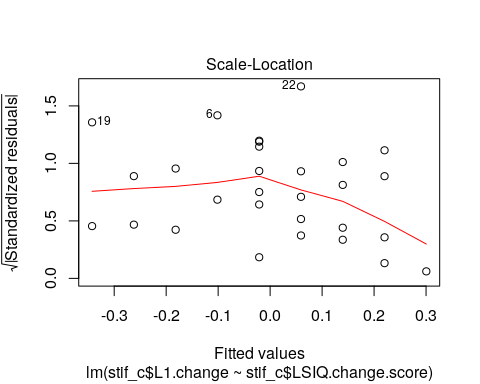

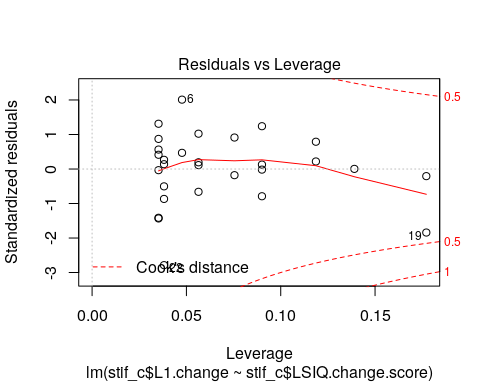


#L2
stif.lm.L2.LSIQ<- lm(stif_c$L2.change~stif_c$LSIQ.change.score)
summary(stif.lm.L2.LSIQ)

##
## Call:
## lm(formula = stif_c$L2.change ~ stif_c$LSIQ.change.score)
##
## Residuals:
## Min 1Q Median 3Q Max
## -1.89514 -0.25138 0.04286 0.40786 1.05811
##
## Coefficients:
## Estimate Std. Error t value Pr(>|t|)
## (Intercept) -0.09436 0.15600 -0.605 0.550
## stif_c$LSIQ.change.score -0.09125 0.06262 -1.457 0.157
##
## Residual standard error: 0.7145 on 27 degrees of freedom
## Multiple R-squared: 0.07292, Adjusted R-squared: 0.03858
## F-statistic: 2.124 on 1 and 27 DF, p-value: 0.1566

plot(stif.lm.L2.LSIQ)


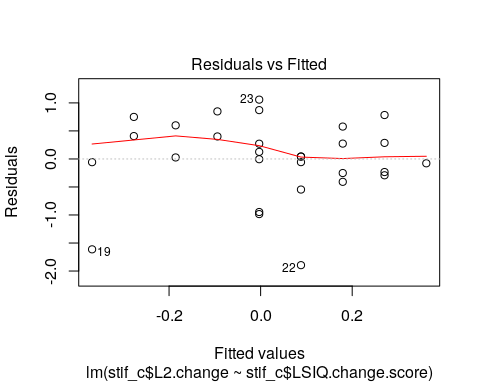

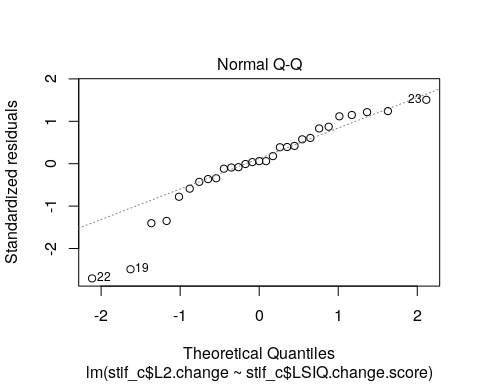

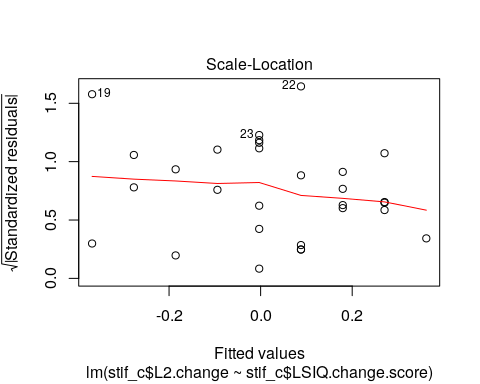

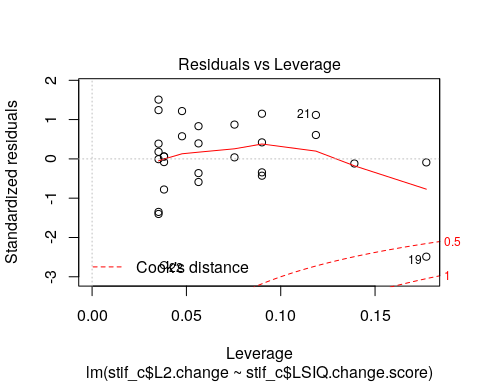


#L3
stif.lm.L3.LSIQ<- lm(stif_c$L3.change~stif_c$LSIQ.change.score)
summary(stif.lm.L3.LSIQ)

##
## Call:
## lm(formula = stif_c$L3.change ~ stif_c$LSIQ.change.score)
##
## Residuals:
## Min 1Q Median 3Q Max
## -1.2780 -0.2937 -0.0011 0.2918 1.0206
##
## Coefficients:
## Estimate Std. Error t value Pr(>|t|)
## (Intercept) -0.02971 0.12730 -0.233 0.817
## stif_c$LSIQ.change.score -0.06209 0.05110 -1.215 0.235
##
## Residual standard error: 0.5831 on 27 degrees of freedom
## Multiple R-squared: 0.05186, Adjusted R-squared: 0.01674
## F-statistic: 1.477 on 1 and 27 DF, p-value: 0.2348

plot(stif.lm.L3.LSIQ)


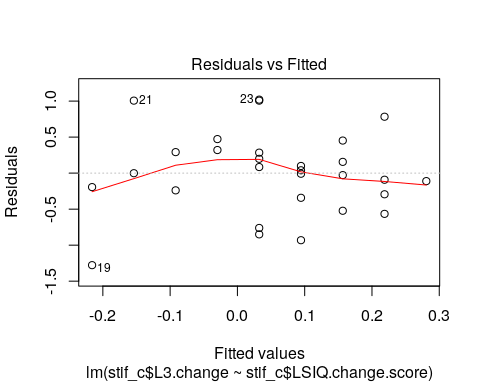

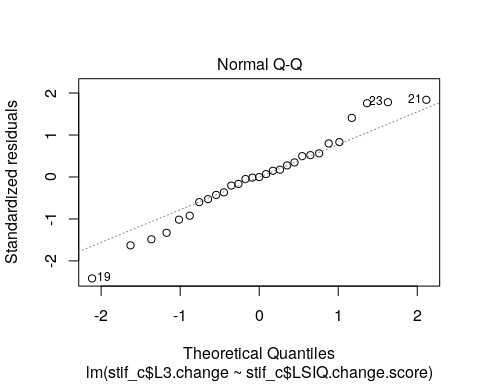

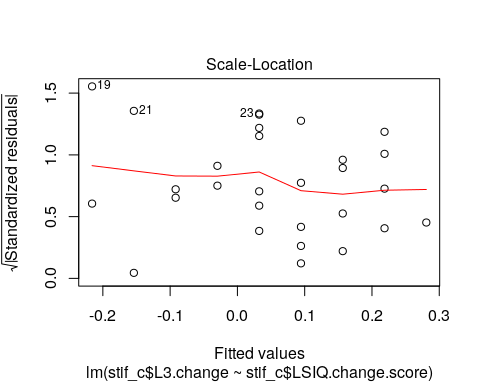

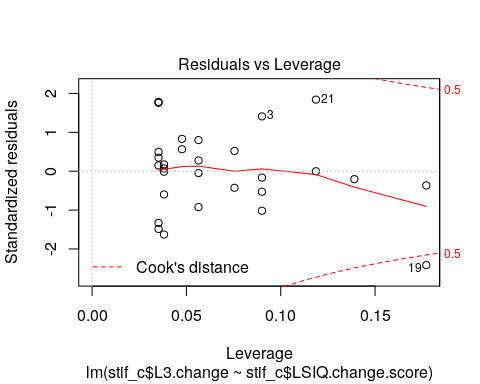


#L4
stif.lm.L4.LSIQ<- lm(stif_c$L4.change~stif_c$LSIQ.change.score)
summary(stif.lm.L4.LSIQ)

##
## Call:
## lm(formula = stif_c$L4.change ~ stif_c$LSIQ.change.score)
##
## Residuals:
## Min 1Q Median 3Q Max
## -1.25108 -0.26808 0.05342 0.36729 1.26242
##
## Coefficients:
## Estimate Std. Error t value Pr(>|t|)
## (Intercept) -0.02154 0.13057 -0.165 0.870
## stif_c$LSIQ.change.score -0.04813 0.05241 -0.918 0.367
##
## Residual standard error: 0.598 on 27 degrees of freedom
## Multiple R-squared: 0.03028, Adjusted R-squared: -0.005632
## F-statistic: 0.8432 on 1 and 27 DF, p-value: 0.3666

plot(stif.lm.L4.LSIQ)


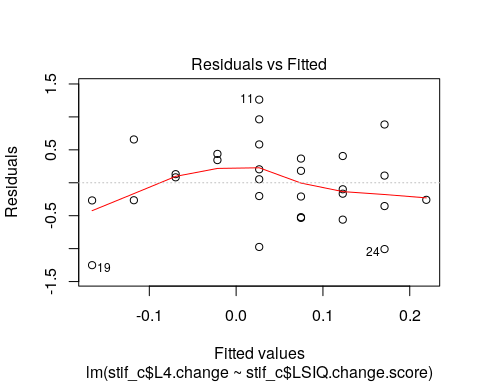

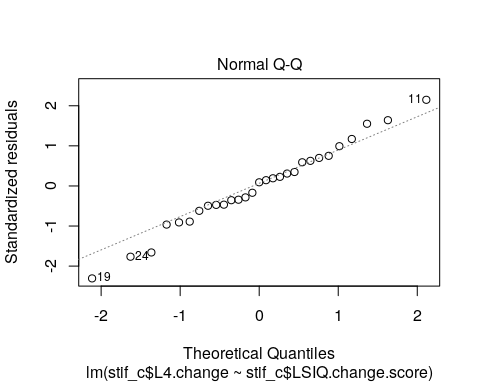

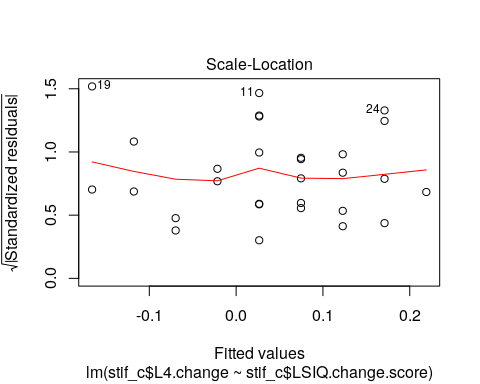

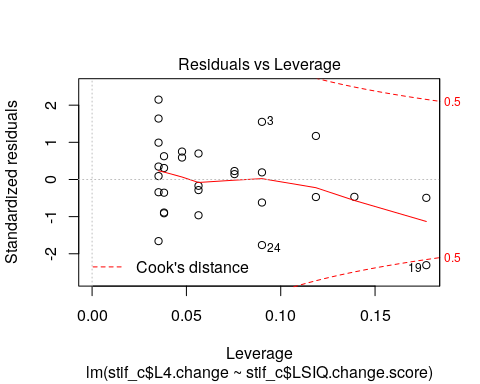


#L5
stif.lm.L5.LSIQ<- lm(stif_c$L5.change~stif_c$LSIQ.change.score)
summary(stif.lm.L5.LSIQ)

##
## Call:
## lm(formula = stif_c$L5.change ~ stif_c$LSIQ.change.score)
##
## Residuals:
## Min 1Q Median 3Q Max
## -1.25370 -0.35743 -0.08526 0.55819 1.80847
##
## Coefficients:
## Estimate Std. Error t value Pr(>|t|)
## (Intercept) -0.02519 0.15086 -0.167 0.869
## stif_c$LSIQ.change.score -0.03472 0.06055 -0.573 0.571
##
## Residual standard error: 0.691 on 27 degrees of freedom
## Multiple R-squared: 0.01203, Adjusted R-squared: -0.02456
## F-statistic: 0.3288 on 1 and 27 DF, p-value: 0.5711

plot(stif.lm.L5.LSIQ)


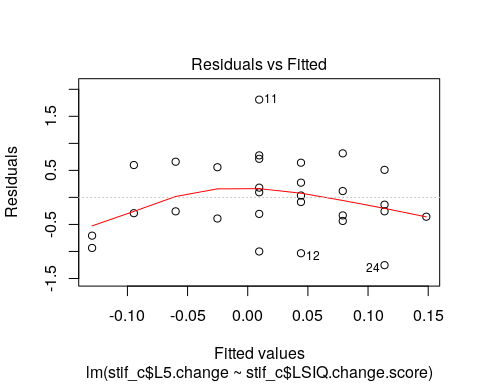

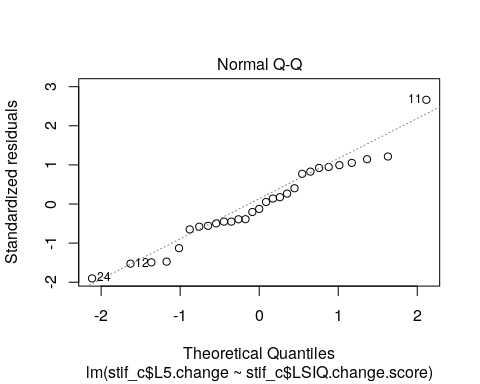

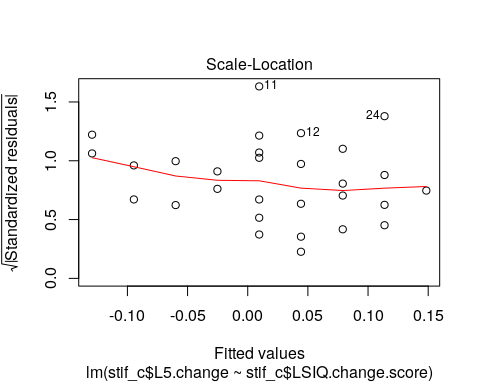

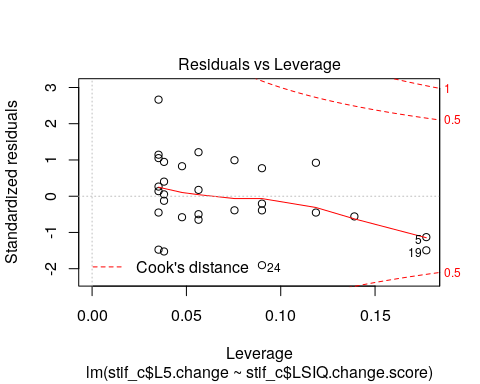


LSIQ plots (regression)

ggplot(stif_c,aes(x=L1.change,y=LSIQ.change.score)) +
 geom_point() +
 geom_abline()


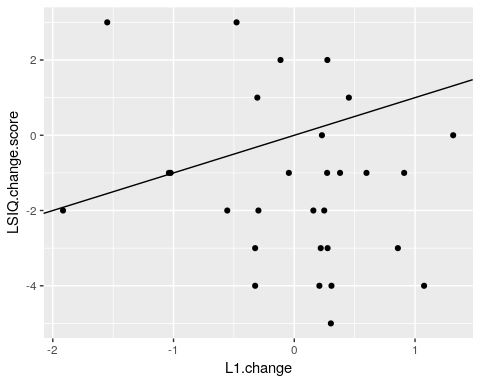


ggplot(stif_c,aes(x=L2.change,y=LSIQ.change.score)) +
 geom_point() +
 geom_abline()


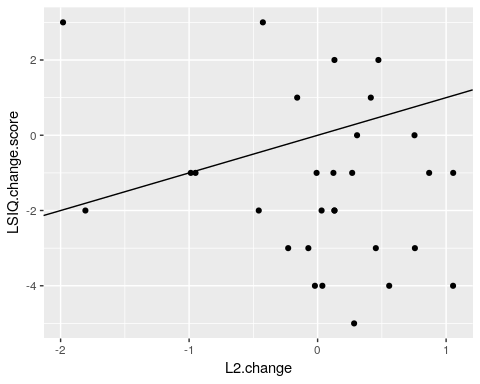


ggplot(stif_c,aes(x=L3.change,y=LSIQ.change.score)) +
 geom_point() +
 geom_abline()


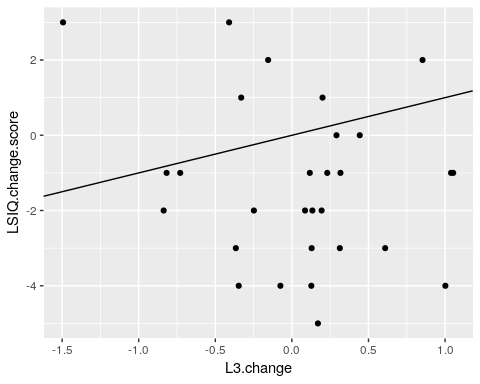


ggplot(stif_c,aes(x=L4.change,y=LSIQ.change.score)) +
 geom_point() +
 geom_abline()


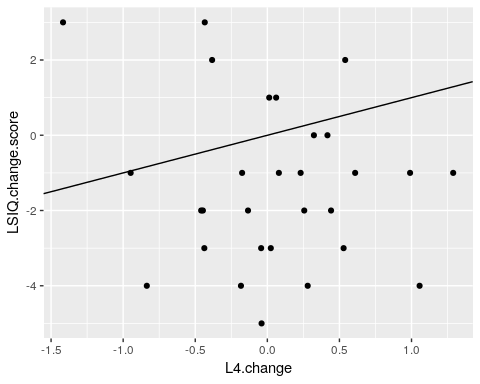


ggplot(stif_c,aes(x=L5.change,y=LSIQ.change.score)) +
 geom_point() +
 geom_abline()


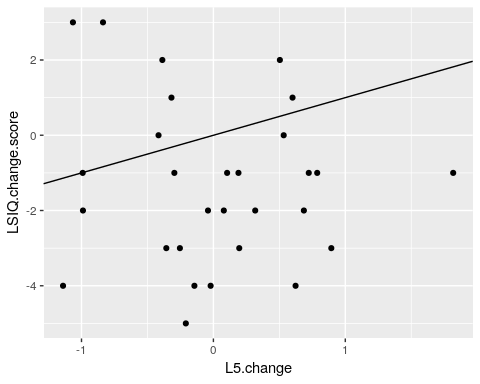


#

# LSDI change

#L1
stif.lm.L1.LSDI<- lm(stif_c$L1.change~stif_c$LSDI.change.score)
summary(stif.lm.L1.LSDI)

##
## Call:
## lm(formula = stif_c$L1.change ~ stif_c$LSDI.change.score)
##
## Residuals:
## Min 1Q Median 3Q Max
## -1.8648 -0.3707 0.1981 0.3222 1.3662
##
## Coefficients:
## Estimate Std. Error t value Pr(>|t|)
## (Intercept) -0.03083 0.15205 -0.203 0.841
## stif_c$LSDI.change.score -0.01939 0.03633 -0.534 0.598
##
## Residual standard error: 0.7398 on 27 degrees of freedom
## Multiple R-squared: 0.01044, Adjusted R-squared: -0.02621
## F-statistic: 0.2848 on 1 and 27 DF, p-value: 0.5979

plot(stif.lm.L1.LSDI)


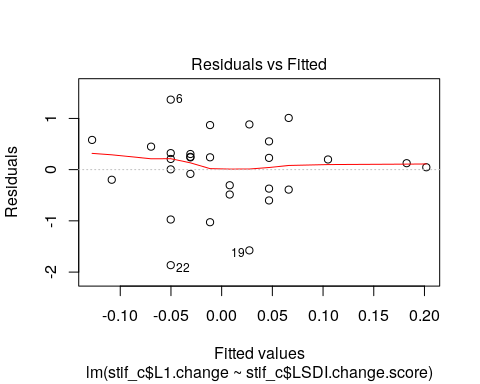

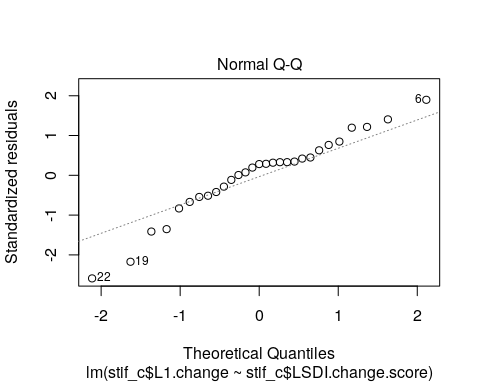

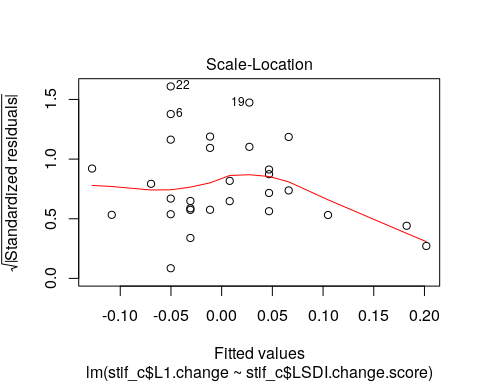

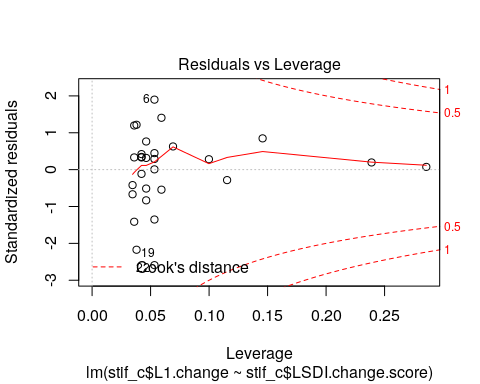


#L2
stif.lm.L2.LSDI<- lm(stif_c$L2.change~stif_c$LSDI.change.score)
summary(stif.lm.L2.LSDI)

##
## Call:
## lm(formula = stif_c$L2.change ~ stif_c$LSDI.change.score)
##
## Residuals:
## Min 1Q Median 3Q Max
## -2.04372 -0.21145 0.09696 0.35920 0.99228
##
## Coefficients:
## Estimate Std. Error t value Pr(>|t|)
## (Intercept) -0.03053 0.15044 -0.203 0.841
## stif_c$LSDI.change.score -0.03108 0.03594 -0.865 0.395
##
## Residual standard error: 0.732 on 27 degrees of freedom
## Multiple R-squared: 0.02695, Adjusted R-squared: -0.00909
## F-statistic: 0.7478 on 1 and 27 DF, p-value: 0.3948

plot(stif.lm.L2.LSDI)


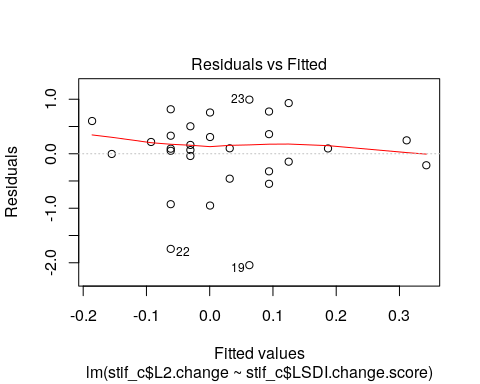

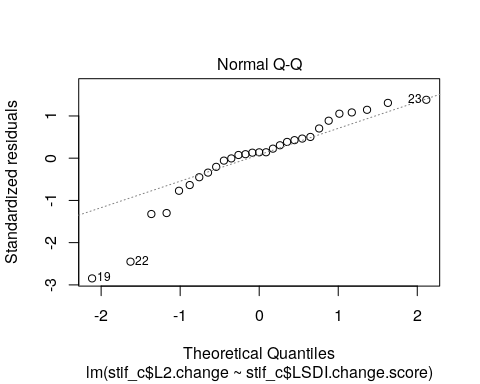

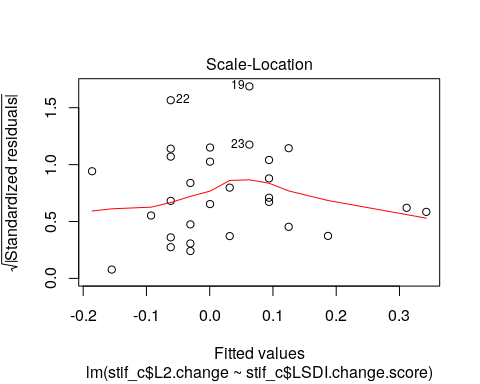

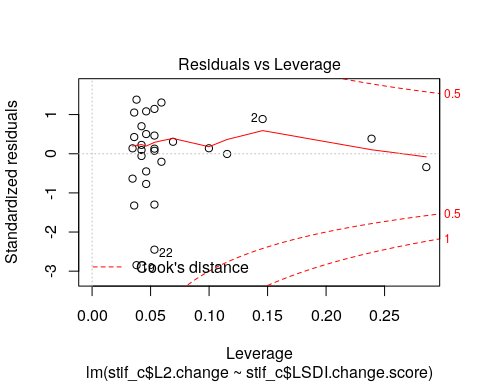


#L3
stif.lm.L3.LSDI<- lm(stif_c$L3.change~stif_c$LSDI.change.score)
summary(stif.lm.L3.LSDI)

##
## Call:
## lm(formula = stif_c$L3.change ~ stif_c$LSDI.change.score)
##
## Residuals:
## Min 1Q Median 3Q Max
## -1.5671 -0.3389 0.1092 0.2692 0.9799
##
## Coefficients:
## Estimate Std. Error t value Pr(>|t|)
## (Intercept) 0.01976 0.12222 0.162 0.873
## stif_c$LSDI.change.score -0.01779 0.02920 -0.609 0.548
##
## Residual standard error: 0.5947 on 27 degrees of freedom
## Multiple R-squared: 0.01356, Adjusted R-squared: -0.02298
## F-statistic: 0.3711 on 1 and 27 DF, p-value: 0.5475

plot(stif.lm.L3.LSDI)


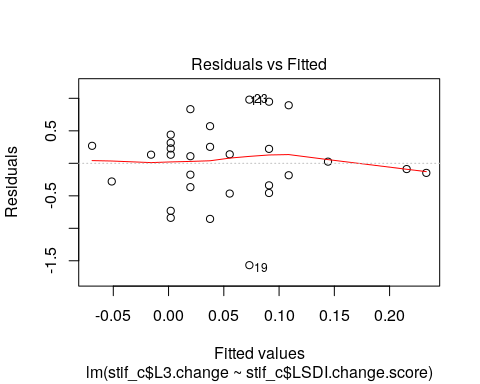

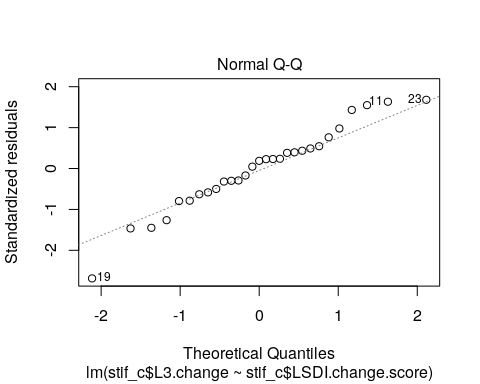

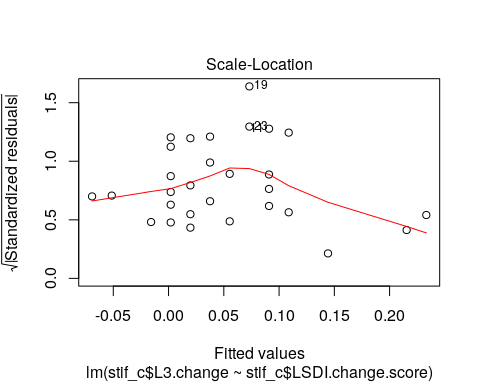

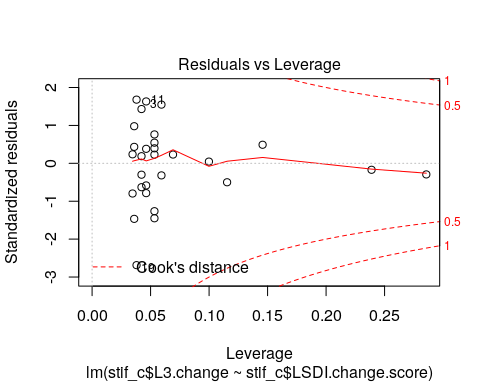


#L4
stif.lm.L4.LSDI<- lm(stif_c$L4.change~stif_c$LSDI.change.score)
summary(stif.lm.L4.LSDI)

##
## Call:
## lm(formula = stif_c$L4.change ~ stif_c$LSDI.change.score)
##
## Residuals:
## Min 1Q Median 3Q Max
## -1.46298 -0.41788 -0.00708 0.38582 1.23931
##
## Coefficients:
## Estimate Std. Error t value Pr(>|t|)
## (Intercept) 0.034881 0.124774 0.280 0.782
## stif_c$LSDI.change.score -0.003701 0.029812 -0.124 0.902
##
## Residual standard error: 0.6071 on 27 degrees of freedom
## Multiple R-squared: 0.0005705, Adjusted R-squared: -0.03645
## F-statistic: 0.01541 on 1 and 27 DF, p-value: 0.9021

plot(stif.lm.L4.LSDI)


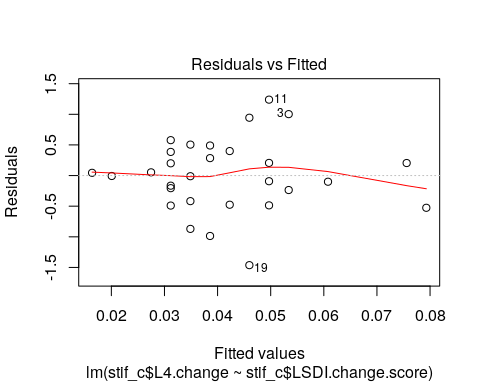

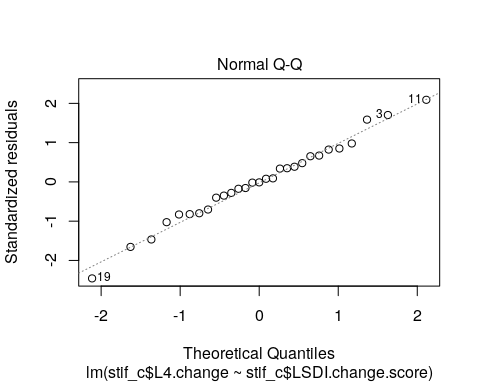

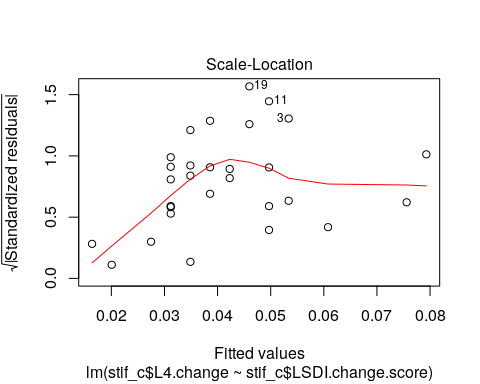

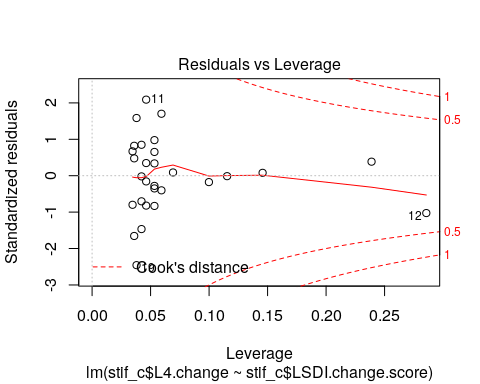


#L5
stif.lm.L5.LSDI<- lm(stif_c$L5.change~stif_c$LSDI.change.score)
summary(stif.lm.L5.LSDI)

##
## Call:
## lm(formula = stif_c$L5.change ~ stif_c$LSDI.change.score)
##
## Residuals:
## Min 1Q Median 3Q Max
## -1.19034 -0.43734 0.01191 0.48267 1.83465
##
## Coefficients:
## Estimate Std. Error t value Pr(>|t|)
## (Intercept) 0.05034 0.14223 0.354 0.726
## stif_c$LSDI.change.score 0.01675 0.03398 0.493 0.626
##
## Residual standard error: 0.6921 on 27 degrees of freedom
## Multiple R-squared: 0.008915, Adjusted R-squared: -0.02779
## F-statistic: 0.2429 on 1 and 27 DF, p-value: 0.6261

plot(stif.lm.L5.LSDI)


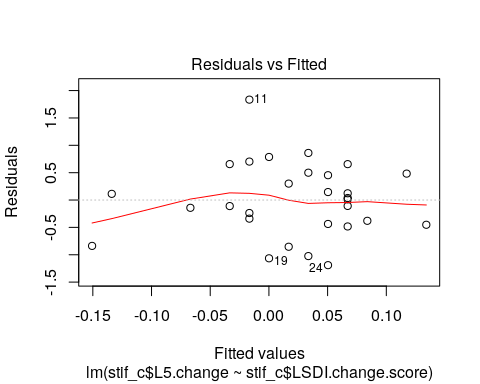

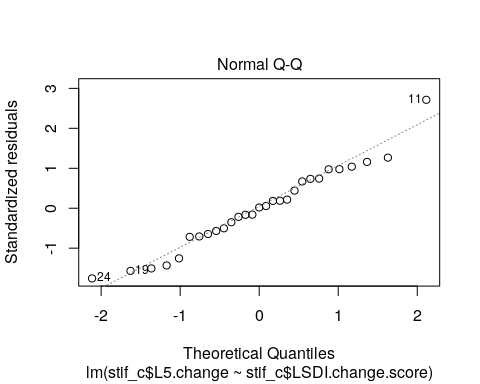

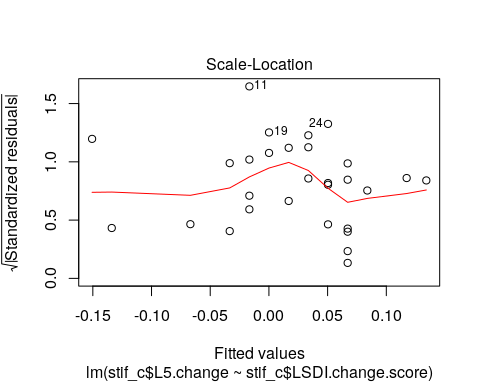

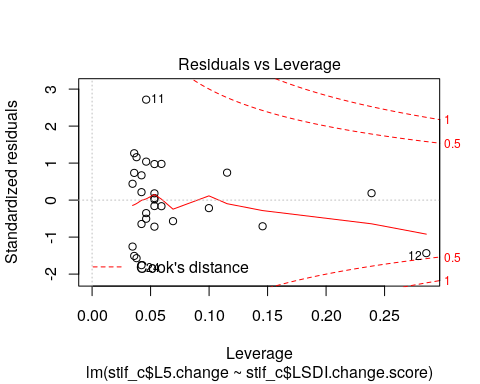


plots

ggplot(stif_c,aes(x=L1.change,y=LSDI.change.score)) +
 geom_point() +
 geom_abline()


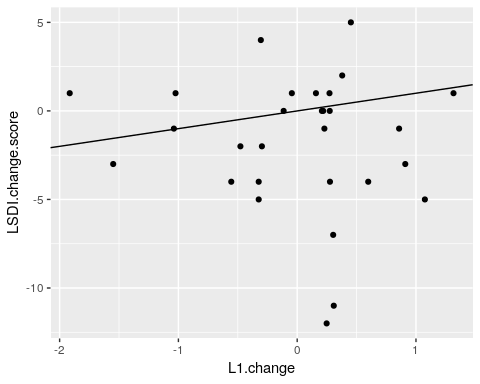


ggplot(stif_c,aes(x=L2.change,y=LSDI.change.score)) +
 geom_point() +
 geom_abline()


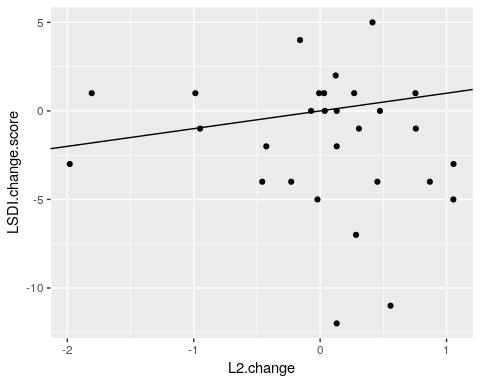


ggplot(stif_c,aes(x=L3.change,y=LSDI.change.score)) +
 geom_point() +
 geom_abline()


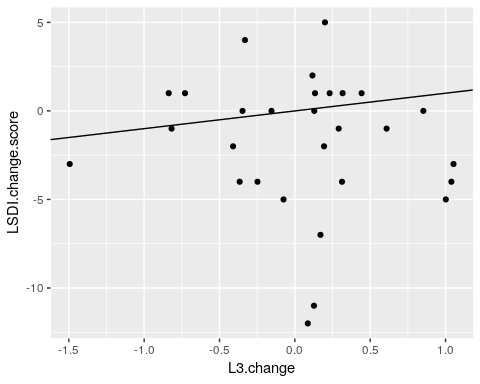


ggplot(stif_c,aes(x=L4.change,y=LSDI.change.score)) +
 geom_point() +
 geom_abline()


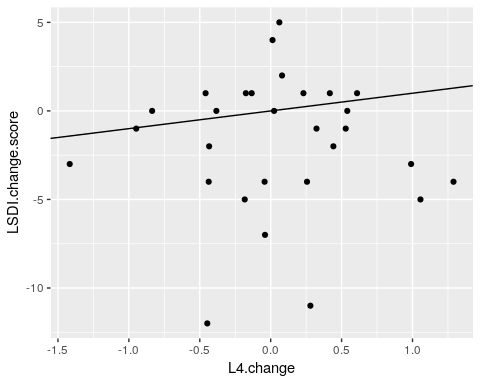


ggplot(stif_c,aes(x=L5.change,y=LSDI.change.score)) +
 geom_point() +
 geom_abline()


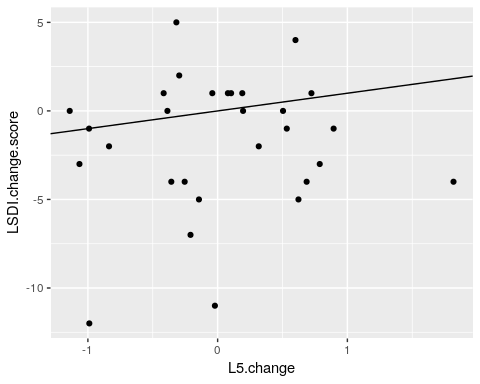


#

# T-test

#L1

t.test(stif_RLSIQ$L1.change,stif_NRLSIQ$L1.change)

##
## Welch Two Sample t-test
##
## data: stif_RLSIQ$L1.change and stif_NRLSIQ$L1.change
## t = 0.1081, df = 11.19, p-value = 0.9158
## alternative hypothesis: true difference in means is not equal to 0
## 95 percent confidence interval:
## -0.6918002 0.7634192
## sample estimates:
## mean of x mean of y
## 0.01380952 -0.02200000

t.test(stif_RLSDI$L1.change,stif_NRLSDI$L1.change)

##
## Welch Two Sample t-test
##
## data: stif_RLSDI$L1.change and stif_NRLSDI$L1.change
## t = 1.416, df = 8.5023, p-value = 0.1924
## alternative hypothesis: true difference in means is not equal to 0
## 95 percent confidence interval:
## -0.2351993 1.0038660
## sample estimates:
## mean of x mean of y
## 0.32200000 -0.06233333

#L2

t.test(stif_RLSIQ$L2.change,stif_NRLSIQ$L2.change)

##
## Welch Two Sample t-test
##
## data: stif_RLSIQ$L2.change and stif_NRLSIQ$L2.change
## t = 0.35006, df = 10.665, p-value = 0.7331
## alternative hypothesis: true difference in means is not equal to 0
## 95 percent confidence interval:
## -0.6314182 0.8691682
## sample estimates:
## mean of x mean of y
## 0.058000 -0.060875

t.test(stif_RLSDI$L2.change,stif_NRLSDI$L2.change)

##
## Welch Two Sample t-test
##
## data: stif_RLSDI$L2.change and stif_NRLSDI$L2.change
## t = 1.8559, df = 10.384, p-value = 0.09204
## alternative hypothesis: true difference in means is not equal to 0
## 95 percent confidence interval:
## -0.0883608 0.9965275
## sample estimates:
## mean of x mean of y
## 0.40100000 -0.05308333

#L3

t.test(stif_RLSIQ$L3.change,stif_NRLSIQ$L3.change)

##
## Welch Two Sample t-test
##
## data: stif_RLSIQ$L3.change and stif_NRLSIQ$L3.change
## t = 0.6307, df = 10.32, p-value = 0.542
## alternative hypothesis: true difference in means is not equal to 0
## 95 percent confidence interval:
## -0.4417159 0.7925611
## sample estimates:
## mean of x mean of y
## 0.1000476 -0.0753750

t.test(stif_RLSDI$L3.change,stif_NRLSDI$L3.change)

##
## Welch Two Sample t-test
##
## data: stif_RLSDI$L3.change and stif_NRLSDI$L3.change
## t = 1.1179, df = 8.0068, p-value = 0.296
## alternative hypothesis: true difference in means is not equal to 0
## 95 percent confidence interval:
## -0.2700516 0.7783849
## sample estimates:
## mean of x mean of y
## 0.262000000 0.007833333

#L4

t.test(stif_RLSIQ$L4.change,stif_NRLSIQ$L4.change)

##
## Welch Two Sample t-test
##
## data: stif_RLSIQ$L4.change and stif_NRLSIQ$L4.change
## t = 0.80989, df = 11.86, p-value = 0.434
## alternative hypothesis: true difference in means is not equal to 0
## 95 percent confidence interval:
## -0.3544075 0.7728837
## sample estimates:
## mean of x mean of y
## 0.0992381 -0.1100000

t.test(stif_RLSDI$L4.change,stif_NRLSDI$L4.change)

##
## Welch Two Sample t-test
##
## data: stif_RLSDI$L4.change and stif_NRLSDI$L4.change
## t = 0.38568, df = 6.0123, p-value = 0.713
## alternative hypothesis: true difference in means is not equal to 0
## 95 percent confidence interval:
## -0.5917222 0.8132888
## sample estimates:
## mean of x mean of y
## 0.13320000 0.02241667

#L5

t.test(stif_RLSIQ$L5.change,stif_NRLSIQ$L5.change)

##
## Welch Two Sample t-test
##
## data: stif_RLSIQ$L5.change and stif_NRLSIQ$L5.change
## t = 0.9746, df = 13.657, p-value = 0.3467
## alternative hypothesis: true difference in means is not equal to 0
## 95 percent confidence interval:
## -0.3223301 0.8569253
## sample estimates:
## mean of x mean of y
## 0.09404762 -0.17325000

t.test(stif_RLSDI$L5.change,stif_NRLSDI$L5.change)

##
## Welch Two Sample t-test
##
## data: stif_RLSDI$L5.change and stif_NRLSDI$L5.change
## t = -0.68945, df = 6.8168, p-value = 0.5133
## alternative hypothesis: true difference in means is not equal to 0
## 95 percent confidence interval:
## -0.9047088 0.4979588
## sample estimates:
## mean of x mean of y
## -0.148000 0.055375

par(mfrow=c(2,2))

# 
