# Supplementary material for: Self-reports vs. physical measures of spinal stiffness
Source: PeerJ. 2020 Dec 7;8:e9598. doi: 10.7717/peerj.9598 (PMC7727369; doi:10.7717/peerj.9598)
Supplement: Supplemental Information 4 [file peerj-08-9598-s004.pdf]

**Lumbar Spine Instability Questionnaire**

1. Jeg føler det som om min ryg "giver efter"
2. Jeg føler behov for ofte at 'knække' i min ryg for at lindre smerten
3. Jeg har ofte tidspunkter hvor min ryg gør ondt, gennem hele dagen
4. Jeg har flere gange før oplevet at min ryg låser når jeg bukke eller bøjer mig
5. Jeg har smerter når sætter mig ned eller rejser mig op
6. Jeg har mange smerter når rejser mig fra liggende stilling, med mindre jeg gør det på den rigtige måde
7. Min smerte forværres nogle gange ved hurtige, uventede eller lettere bevægelser
8. Jeg har problemer med at sidde uden ordentlig støtte som i en stol og har det bedre med en lændestøtte
9. Jeg tolererer ikke vedvarende stillinger hvor jeg ikke kan bevæge mig
10. Det synes som om min tilstand bliver værre med tiden
11. Jeg har haft dette problem i lang tid
12. Det lindrer nogle gange midlertidigt når jeg bruger et lændebælte eller støtte
13. Jeg får muskel spændinger ved mange lejligheder
14. Nogle gange er jeg bekymret for at bevæge mig pga mine smerter
15. Jeg har tidligere fået en rygskade pga et traume/en ulykke

Svar:
JA
NEJ

**Lumbar Stiffness Disability Index**

1. Bøje dig ned til fødderne for selv at tage undertøj og bukser på
2. Bøje i lænden for at tage strømper og sko på
3. Føre et køretøj
4. Udføre personlige hygiejne efter toilet besøg
5. Bøje forover for at samle småting op fra gulvet
6. Komme ind og ud af sengen
7. Sætte og rejse sig fra en stol
8. Vaske den nedre del af kroppen
9. Stige ind og ud af et køretøj
10. Have samleje

Svar:
0 - Overhovedet ingen betydning
1 - Mindre betydning
2 - Nogen betydning
3 - Behov for hjælp
4 - Umuliggjort
